# Cleavage of Cartilage Oligomeric Matrix Protein (COMP) by ADAMTS4 generates a neoepitope associated with osteoarthritis and other forms of degenerative joint disease

**DOI:** 10.1016/j.matbio.2024.12.005

**Published:** 2024-12-11

**Authors:** Rens de Groot, Patricia Badía Folgado, Kazuhiro Yamamoto, Daniel R. Martin, Christopher D. Koch, Danielle Debruin, Sophie Blagg, Alexander F. Minns, Sumit Bhutada, Josefin Ahnström, Jonathan Larkin, Anders Aspberg, Patrik Önnerfjord, Suneel S. Apte, Salvatore Santamaria

**Affiliations:** a Institute of Cardiovascular Science, University College London, 51 Chenies Mews, London WC1E 6HX, United Kingdom; b Department of Immunology and Inflammation, Imperial College London, Du Cane Road, London W12 0NN, United Kingdom; c Institute of Life Course and Medical Sciences, Faculty of Health and Life Sciences, University of Liverpool, 6 West Derby Street, Liverpool L7 8TX, United Kingdom; d Department of Biomedical Engineering, Cleveland Clinic Lerner Research Institute, Cleveland, OH 44195, USA; e Department of Biochemical Sciences, School of Biosciences, Faculty of Health and Medical Sciences, Edward Jenner Building, University of Surrey, Guildford, Surrey GU2 7XH, United Kingdom; f SynOA Therapeutics, Philadelphia, PA, USA; g Research Unit of Health Sciences and Technology, Faculty of Medicine, University of Oulu, Oulu, Finland; h Rheumatology and Molecular Skeletal Biology, Department of Clinical Sciences Lund, Lund University, Lund, Sweden

**Keywords:** Cartilage oligomeric matrix protein, ADAMTS, ADAMTS4, Osteoarthritis, Neoepitope, Extracellular matrix, Proteolysis, Biomarker

## Abstract

Osteoarthritis (OA) is a highly prevalent joint disease, affecting millions of people worldwide and characterized by degradation of articular cartilage, subchondral bone remodeling and low-grade inflammation, leading to pain, stiffness and disability. Cartilage Oligomeric Matrix Protein (COMP) is a major structural component of cartilage and its degradation has been proposed as a marker of OA severity/progression. Several proteases cleave COMP *in vitro*, however, it is unclear which of these COMPase activities is prevalent in an osteoarthritic joint. Here, using purified recombinant proteins, we show that A Disintegrin And Metalloproteinase with Thrombospondin motifs 4 (ADAMTS4) is the most potent COMPase, followed by ADAMTS1. Using liquid chromatography-tandem mass spectrometry, we identified several novel cleavage sites in COMP resulting from ADAMTS4 and ADAMTS1 activity. Cleavage at S^77^-V^78^ disrupted the pentameric organization of COMP and generated a neopeptide previously identified in the synovial fluid of OA patients. Immunoblots with anti-QQS^77^ antibodies confirmed that ADAMTS4 efficiently cleaved this peptide bond. By analyzing five ADAMTS4 variants, we found that the C-terminal spacer domain is strictly necessary for COMPase activity and identified the specific residues involved in the interaction with COMP. An inhibitory anti-ADAMTS4 antibody significantly decreased generation of the COMP QQS^77^ neoepitope in human OA cartilage explants, implicating ADAMTS4 as a key protease in generating the QQS^77^ neopeptides in OA. Since another major ADAMTS4 substrate is aggrecan, the most abundant proteoglycan in cartilage, these findings highlight that, by cleaving both COMP and aggrecan, ADAMTS4 may play a crucial role in modulating the structural integrity of cartilage.

## Introduction

Osteoarthritis (OA) is the most common chronic joint disease and a leading cause of pain and disability worldwide, affecting all synovial joints, most commonly the hips, knees, hands and spine [1]. Degradation of articular cartilage is an important feature of this multifactorial disease which also affects the synovium, subchondral bone, tendons and ligaments [2,3]. While the incidence of OA is rising due to an increase in life expectancy and obesity, current medical treatments are limited to symptomatic management and do not correct the underlying structural alterations of the joint or improve function [4].

**Fig. 1.**
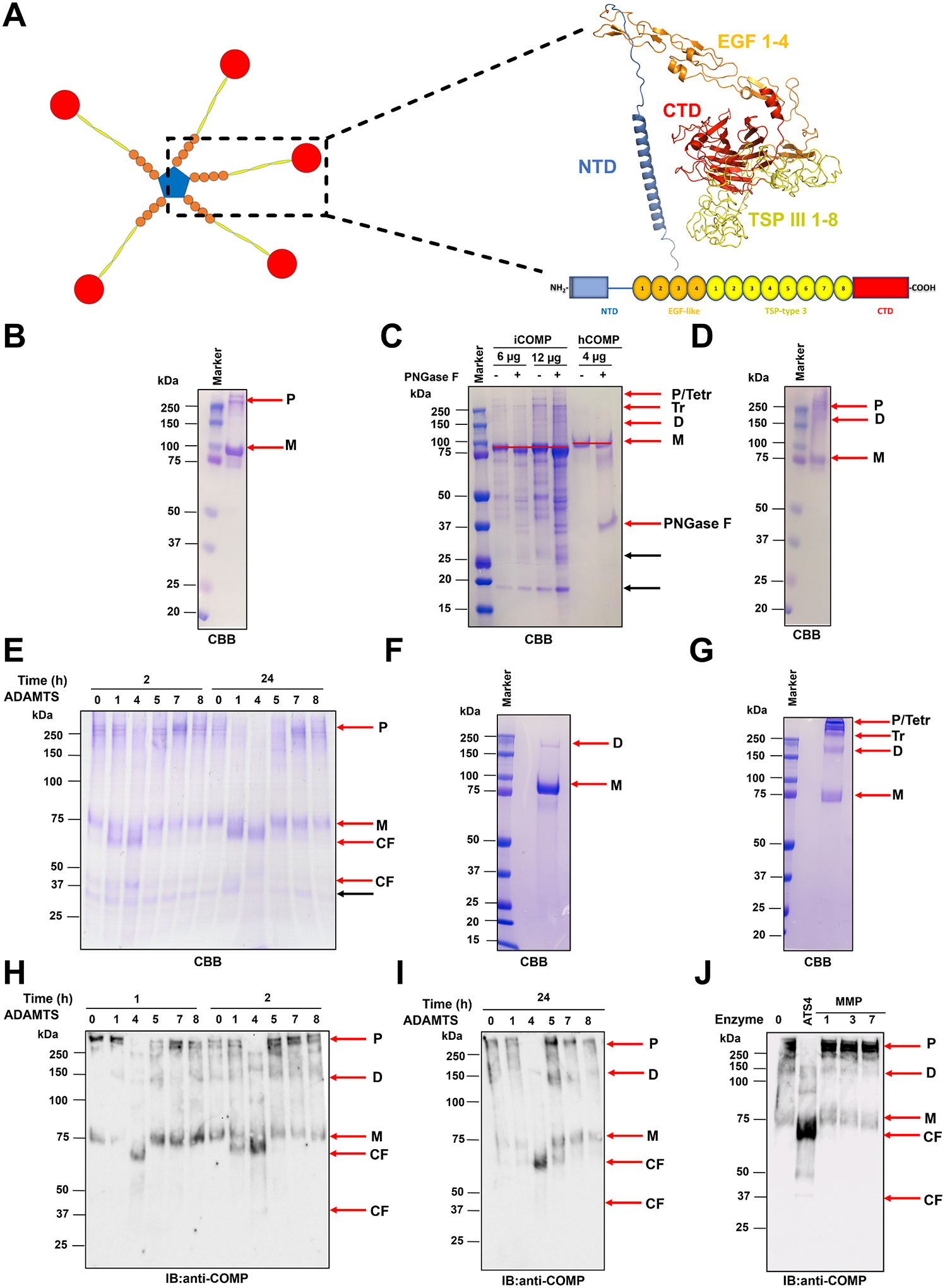
COMPase activity of selected ADAMTS proteases. **A)** Schematic of pentameric COMP (left), domain organization and 3D model (AlphaFold ID: AF-P49747-F1) of the monomer (right). CTD, C-terminal domain; EGF, epidermal-growth factor-like repeats; NTD, N-terminal domain; TSP, thrombospondin type III repeat. **B-D)** Coomassie Brilliant Blue (CBB) staining of purified iCOMP (4 μg/lane) under reducing (5 % β-mercaptoethanol, **B and C**) and non-reducing (**D**) conditions. **C)** To detect N-linked glycosylation, iCOMP and hCOMP were treated with (+) or without (−) PNGase F (1 mU/μL) for 16 h at 37 °C before reducing SDS-PAGE (4–12 %) and CBB. The red line highlights the shift in molecular weight upon PNGase F treatment. The concentration of PNGase F was the same in all samples (see Experimental Procedures); therefore, since different volumes of samples were loaded on the gel, the intensity of the PNGase F band at ~37 kDa varied with different COMP amounts. The gel is representative of two independent experiments. **E)** Digestion of iCOMP by ADAMTS proteases. iCOMP (520 nM) was incubated with ADAMTS1, 4, 5, 7 and 8 (12 nM) or buffer alone (0) for 2 h and 24 h at 37 °C. Samples were then subjected to SDS-PAGE under non-reducing conditions and CBB staining. The gel is representative of two independent experiments. **F, G)** CBB staining of purified hCOMP (4 μg/lane) under reducing (F) and non-reducing (G) conditions. **H, I)** Digestion of hCOMP by ADAMTS proteases. hCOMP (520 nM) was incubated with ADAMTS1, 4, 5, 7 and 8 (12 nM) or buffer alone (0) for 1 h, 2 h (**H**), and 24 h **(I)** at 37 °C. Samples were then subjected to SDS-PAGE under non-reducing conditions and probed with a polyclonal anti-COMP antibody. The blot is representative of 3 independent experiments. **J)** Comparison between the COMPase activity of ADAMTS4 (ATS4) and that of MMP1, MMP3, and MMP7. hCOMP (520 nM) was incubated with each protease (12 nM) or buffer alone (0) for 2 h at 37 °C. Samples were then subjected to SDS-PAGE under non-reducing conditions and probed with a polyclonal anti-COMP antibody. The blot is representative of two independent experiments. CF, cleavage fragment; D, dimer; IB, immunoblot; M, monomer; P, pentamer; Tr, trimer; Tetr, tetramer. Black arrows indicate degradation products originated during expression and purification of iCOMP.

The articular cartilage extracellular matrix (ECM) comprises several collagens, principally collagen II, proteoglycans, of which aggrecan is most abundant, and several non-collagenous, non-proteoglycan macromolecules contributing to ECM structure and regulation [2,5]. Cartilage Oligomeric Matrix Protein (COMP), also named thrombospondin 5, is an abundant cartilage component [6], also expressed in synovium [7], tendons [8], and vascular beds [9,10]. COMP is synthesized and assembled intracellularly as a homopentamer with a molecular weight of 524 kDa and secreted into the ECM [11]. Each monomer contains a coiled-coil N-terminal domain (NTD), necessary for pentamer assembly, 4 epidermal-growth factor (EGF)-like repeats, 8 thrombospondin type III (TSP) repeats and a C-terminal globular domain (CTD, Fig. 1A). In cartilage ECM, COMP interacts directly with collagens I, II, IX, XII, and XIV [12,13], matrilin 3 [14], fibronectin [15] and aggrecan [16].

Autosomal dominant missense and in-frame insertion/deletion mutations in the *COMP* gene cause two forms of skeletal dysplasia: pseudoachondroplasia [17–19] and multiple-epiphyseal dysplasia [13,19]. Both conditions are characterized by severe to mild short-limb dwarfism with early-onset OA [20]. A third *COMP* variant (D369H) was identified in a genome-wide association study as a risk factor for hip replacement [21]. These clinical associations reflect the key role of COMP in skeletal development and articular cartilage biology. Mice bearing a pseudochondroplasia mutation in COMP also develop short-limb dwarfism, but *Comp*-deficient mice do not [22,23]. Following these findings, it was proposed that the pathogenic mutations caused misfolding of the protein, reducing secretion by chondrocytes and affecting bone growth. *Comp*-deficient mice were more susceptible to inflammatory arthritis [24], suggesting that COMP may be protective against inflammation.

COMP is a well-established marker of cartilage breakdown in OA [25]. Elevated serum COMP is associated with early-stage OA [26–34], and its levels are increased following exercise [35], in obesity [36], and after traumatic knee injury [37,38]. COMP fragments are released from the ECM into the circulation following localized proteolytic events but the identity of the protease(s) responsible for generating these COMP fragments has not been conclusively established.

Several proteases were implicated in COMP degradation and turnover, including members of the matrix metalloproteinase (MMP) [39–41] and A Disintegrin And Metalloproteinase with Thrombospondin motifs (ADAMTS) family of zinc metalloproteinases [41–44]. The 19 ADAMTS proteases are all secreted and essential for numerous developmental and homeostatic processes in humans and other animal species [45]. They have a prodomain, a metalloproteinase domain (Mp), a disintegrin-like domain (Dis), a central thrombospondin type 1 (TSR) repeat, and a cysteine-rich (CR) domain followed by a spacer (Sp) domain. ADAMTS4 contains only these domains, but other ADAMTS proteases have additional C-terminal ancillary domains. ADAMTS4, ADAMTS7 and ADAMTS12 were previously reported to cleave COMP with different catalytic efficiency *in vitro* [42–44], but so far this activity has not been functionally linked to the specific cleavage sites observed in OA patients. Here, we compare the COMPase activity of selected ADAMTS proteases and highlight a central role for ADAMTS4 in COMP breakdown in human OA.

## Results

### Comparison of COMPase activity of MMP and ADAMTS family members

For functional characterization of COMPase activity, we initially expressed human full-length COMP with C-terminal V5 and 6x His tags in *Drosophila melanogaster* Schneider cells 2 (S2). Following Ni^2+^-chromatography, high amounts of purified COMP were obtained (final yield: 4.4 mg/L of conditioned medium). When subjected to SDS-PAGE under reducing conditions (5 % β-mercaptoethanol) and Coomassie brilliant blue (CBB) staining, purified *Drosophila melanogaster* S2-expressed COMP (iCOMP) migrated as a major band at ~100 kDa, corresponding to the monomeric form (Fig. 1B), as previously observed [11,46,47]. This is higher than the predicted molecular mass of 82.4 kDa, most likely due to *N*-glycosylation [11,46]. To confirm the presence of *N*-glycans, iCOMP was treated with Peptide *N*-glycosidase F (PNGase F) prior to SDS-PAGE under reducing conditions and CBB staining (Fig. 1C). A lower apparent mass shift in molecular weight was observed similar to that predicted from the primary sequence, confirming modification with one or more *N*-linked glycans. Under non-reducing conditions, COMP is expected to run as a pentamer, but we observed a mixture of monomers, dimers, trimers, tetramers and pentamers (~100–500 kDa) (Fig. 1D), as noted previously [13], likely resulting from fragmentation. Prolonged (16 h) incubation of purified iCOMP at 37 °C resulted in the appearance of additional bands, possibly due to digestion by contaminant protease (s) (compare Fig. 1C with Fig. 1B). Nevertheless, we initially used iCOMP to assess the COMPase activity of different ADAMTS proteases using non-reducing conditions to better distinguish the pentameric protein from monomers and fragments. iCOMP was incubated with recombinant purified ADAMTS1, 4, 5, 7, and 8 and the digestion products were analyzed by non-reducing SDS-PAGE and CBB staining (Fig. 1E). All ADAMTS proteases used in this set of experiments were full-length, the only exception being ADAMTS7, which was truncated before the C-terminal protease and lacunin (PLAC) domain [48,49]. As a control, iCOMP was incubated in the absence of proteases (buffer only). Specific degradation products at ~70 kDa appeared after 2 h incubation of iCOMP with ADAMTS1 and ADAMTS4, which markedly increased after 24 h, at which point pentameric iCOMP was almost completely proteolyzed. No detectable cleavage was observed by ADAMTS5, 7, and 8, even after 24 h incubation. In the presence of ADAMTS4, an additional band at ~40 kDa appeared after 2 h incubation and was less intense after 24 h (Fig. 1E).

Although the *Drosophila melanogaster* S2 cell expression system provided high yields of purified COMP, glycosylation in insect cells differs from mammalian cells. For instance, the predominant insect *N*-glycan, the fucosylated Man3GlcNAc2Fuc paucimannose hexasaccharide, lacks the structural and conformational heterogeneity typical of mammalian *N*-glycans [50] which could potentially affect susceptibility to cleavage. Additionally, as mentioned above, prolonged incubation of purified iCOMP at 37 °C resulted in the appearance of nonspecific bands, possibly due to trace amounts of contaminating proteases (Fig. 1C). To address these issues, COMP expression was undertaken in a mammalian cell system.

COMP cDNA was transiently expressed in HEK293-F cells and is hereafter referred to as hCOMP (please note that both iCOMP and hCOMP constructs express human full-length COMP protein). hCOMP was purified from the medium as before [51] and run on SDS-PAGE under both reducing and non-reducing conditions (Fig. 1F and 1G). Overall, the migration pattern was similar to that of iCOMP. Treatment of hCOMP with PNGase F confirmed the presence of N-glycans (Fig. 1C). No additional bands were observed upon prolonged (16 h) incubation of purified hCOMP at 37 °C (Fig. 1C), suggesting that no contaminant protease activity was present in this preparation and/or the longer glycans shield proteolytically susceptible peptide bonds. We then assessed the COMPase activity of ADAMTS proteases against hCOMP. hCOMP was digested with recombinant ADAMTS1, 4, 5, 7, and 8 for 1 h, 2 h (Fig. 1H) and 24 h (Fig. 1I). Since lower yields of hCOMP (0.24 mg/L conditioned medium) did not allow us to load enough material to visualize low-intensity cleavage fragments on SDS-PAGE using CBB, the digests were analyzed by immunoblot using a polyclonal anti-COMP antibody. The digestion patterns replicated those observed for iCOMP (Fig. 1E), thus showing that differences in glycosylation between the two expression systems did not affect proteolytic susceptibility to ADAMTSs. A prominent band of ~70 kDa appeared within 1 h of digestion when COMP was incubated with ADAMTS4, but not ADAMTS1; after 2 h, the same band appeared also in the presence of ADAMTS1 and, after 24 h, in the presence of ADAMTS5 (Fig. 1I). The appearance of this band was accompanied by reduced intensity of the intact COMP pentamer. The cleavage product disappeared following prolonged (24 h) incubation with ADAMTS1 (Fig. 1I), suggesting the occurrence of additional cleavage events which generated smaller fragments and may have destroyed the epitopes recognized by the antibody. Importantly, the ~70 kDa band was not present when COMP was incubated in the presence of buffer, ADAMTS7 or ADAMTS8, even after 24 h digestion (Fig. 1I). The size of the ~70 kDa cleavage fragment, close to that of monomeric COMP, combined with a reduction in the pentamer levels, suggests that the cleavage site is near or within the NTD and so disrupts pentamerization (Fig. 1A). As observed for iCOMP (Fig. 1E), a second cleavage fragment at ~40 kDa was generated by ADAMTS4 after 2 h incubation (Fig. 1H–J), with its intensity decreasing after 24 h (Fig. 1I). The lack of detectable COMPase activity by ADAMTS7 under these conditions was surprising, considering COMP was the first reported substrate for this protease [43]. We repeated this assay in the presence of 2 mM ZnCl_2_, which was reported to enhance ADAMTS7 COMPase activity [43] but were also under these conditions unable to detect any ADAMTS7-generated COMP fragments (Supplementary Fig. 1A). We found that 2 mM ZnCl_2_ abrogated ADAMTS4 COMPase activity, as previously shown for other metalloproteinases [52,53]. Based on these results, we aimed to compare ADAMTS4 COMPase activity with that of MMP3, known to release COMP cleavage fragments from articular cartilage [39], alongside MMP1 and MMP7 whose activity against COMP was not previously investigated. None of these MMPs cleaved COMP after 2 h incubation at the same concentrations used for ADAMTS4 (Fig. 1J). Taken together, these results suggest that ADAMTS4 is the most active COMPase among the metalloproteinases analyzed under these conditions, followed by ADAMTS1 and ADAMTS5.

### Identification of ADAMTS4 and ADAMTS1 cleavage sites in COMP

Since ADAMTS4 and ADAMTS1 exhibited the highest COMPase activity among the metalloproteinases tested, we set out to identify which peptide bond(s) in COMP they cleaved, using a recently described label-free quantitative proteomics approach [54]. Purified hCOMP was incubated with recombinant human ADAMTS4 and ADAMTS1 for 2 h, based on immunoblot data indicating several discrete ADAMTS-generated COMP fragments but not over-digestion at this time point (Fig. 1H). As controls, similar reactions were set up with ADAMTS4 or ADAMTS1 inactive variants (with mutation E→Q at residues 362 and 402 and designated as ADAMTS4 or ADAMTS1 EQ, respectively). The reactions were subsequently digested with trypsin followed by liquid chromatography-tandem mass spectrometry (LC-MS/MS). We first determined ratios of peptide abundance in digests with active (wild type, WT) enzyme and inactive (EQ) control (WT/EQ). Next, *z*-scores were calculated for each LC-MS/MS experiment to define outlier ratios indicative of ADAMTS4 or ADAMTS1-mediated proteolysis. In addition to semi-tryptic peptides with significant *z*-scores, the data were analyzed manually to identify tryptic peptides spanning the putative cleavage sites. A converse ratio of tryptic peptides relative to that of semi-tryptic peptides provided supporting evidence for cleavages at the site.

**Fig. 2.**
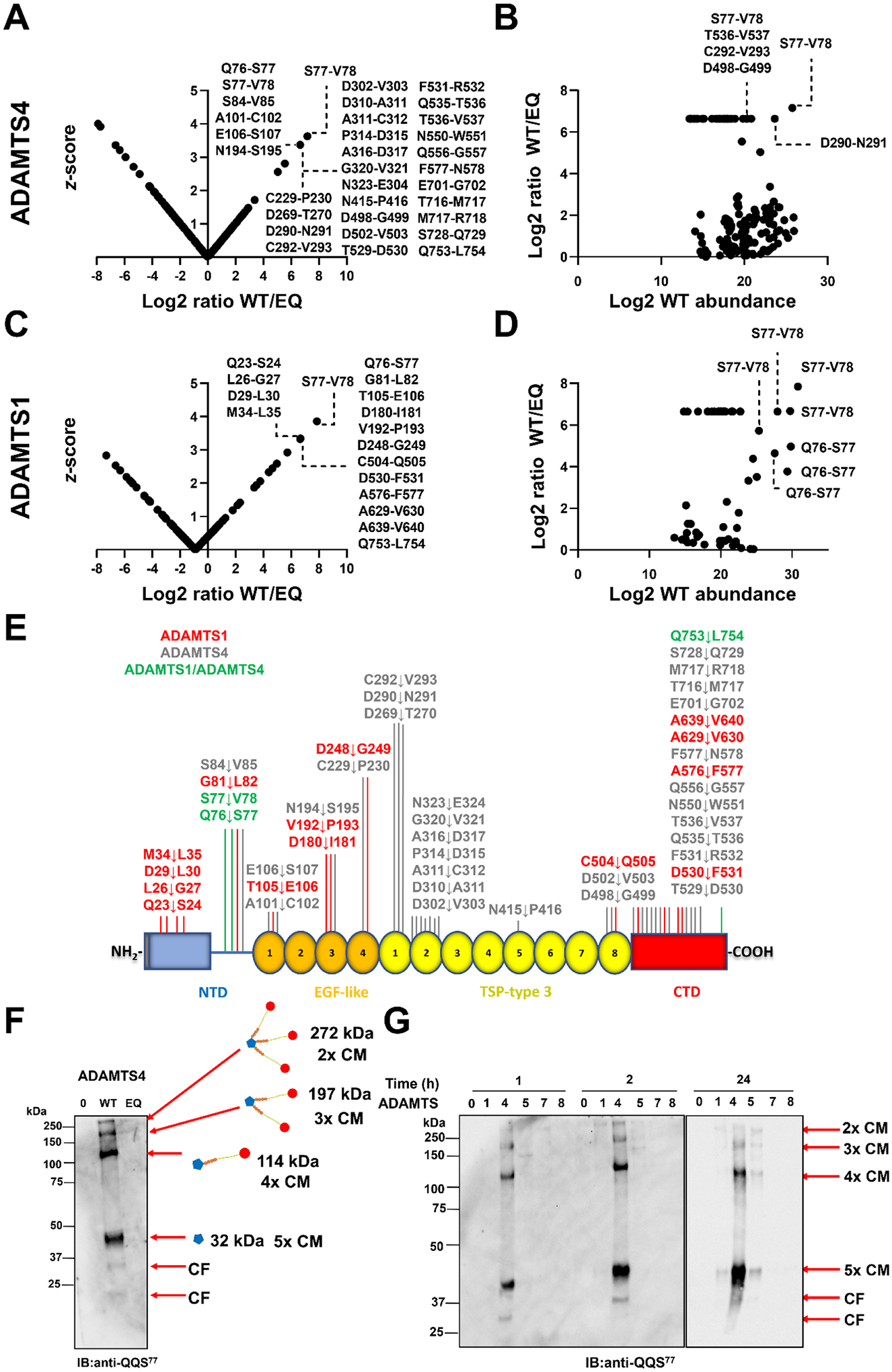
Identification of ADAMTS4 and ADAMTS1 cleavage sites in COMP. hCOMP was digested with ADAMTS4 **(A, B)**, and ADAMTS1 (**C, D**) (each at 12 nM) for 2 h at 37 °C. Panels A and C show the *z*-score of semi-tryptic peptides having higher abundance in the digests with the WT proteases whereas panels B and D show the log_2_ abundance and log_2_ ratio of the *z*-score of significant semi-tryptic peptides in the WT protease digests. Peptides only found in the WT were given an arbitrary ratio of 100 (log_2_100= 6.64). **E)** Overview of ADAMTS cleavage sites in COMP identified by the *z*-score method shown on a graphic representation of COMP. **F)** ADAMTS4 cleaves COMP at the S^77^-V^78^ bond. hCOMP (520 nM) was incubated either with ADAMTS4 WT, ADAMTS4 EQ (each at 12 nM) or buffer alone (0) for 2 h at 37 °C. Samples were then subjected to SDS-PAGE under non-reducing conditions for immunoblotting with an anti-QQS^77^ antibody. **G)** COMPase activity of ADAMTS proteases at the S^77^-V^78^ bond. hCOMP (520 nM) was incubated with ADAMTS1, ADAMTS4, ADAMTS5, ADAMTS7, and ADAMTS8 (each at 12 nM) or buffer alone (0) for the indicated periods of time at 37 °C. Samples were then subjected to SDS-PAGE under non-reducing conditions for immunoblotting with an anti-QQS^77^ antibody. The different cleavage fragments are reported as cartoons alongside the expected molecular weight and the number of cleaved monomers (CM). The vertical line indicates where lanes were digitally rearranged from original blots for clarity of presentation. Immunoblots are representative of two independent experiments. CF, unidentified cleavage fragments, most likely representing further degradation products of the 32 kDa species (fully cleaved NTD). IB, immunoblot.

Of 302 semi-tryptic peptides identified in ADAMTS4 digests, 35 peptides were only found in the presence of WT ADAMTS4 (these are referred to as singletons) and 2 additional semi-tryptic peptides had a significant (>2) z-score, for a total of 37 peptides putatively resulting from ADAMTS4 cleavage. From these peptides, 32 unique potential cleavage sites were identified (Fig. 2A, B, Supplementary Fig. 2 and Table 1). Cleavage at S^77^-V^78^ in the NTD was identified by 4 semi-tryptic peptide singletons in WT ADAMTS4 digests, supported by a corresponding tryptic peptide singleton in ADAMTS4 EQ (Supplementary Fig. 3A). Semi-tryptic peptides resulting from cleavage at S^77^-V^78^ were among the most abundant in WT digests based on peptide intensity (Fig. 2B). Moreover, cleavage at S^77^-V^78^ is predicted to generate a C-terminal fragment matching in size the major 70 kDa band observed when hCOMP was incubated with either ADAMTS4 or ADAMTS1 for 2 h and probed by a polyclonal COMP antibody (Fig. 1H). The adjacent cleavage site Q^76^-S^77^ was identified by one semi-tryptic peptide and supported by a tryptic peptide present exclusively in the WT and EQ ADAMTS4 digests, respectively. These data suggest that S^77^-V^78^ in the NTD could be a preferred cleavage site with additional cleavage occurring one residue upstream at Q^76^-S^77^.

Several other potential cleavage sites were supported by an increased semi-tryptic peptide abundance with a concomitant reduction in abundance of the overlapping tryptic peptide (Table 1). We also detected 5 tryptic peptides with significant *z*-scores in the WT ADAMTS4 digests (Supplementary Fig. 3A), which could indicate trypsin-like cleavages, and 90 semi-tryptic peptides with high z-scores in the EQ digests whose origins are unclear (Fig. 2A).

Of 278 semi-tryptic peptides observed in the ADAMTS1 digests of COMP (Fig. 2C, Supplementary Fig. 3B and Table 2), 22 were singletons in WT ADAMTS1 digests and 5 had a significant *z*-score, for a total of 27 peptides putatively resulting from ADAMTS1 cleavage. From these peptides, 17 unique potential cleavage sites were identified (Fig. 2C–E, Supplementary Fig. 4, and Table 2). Thirteen tryptic COMP peptides were detected only in ADAMTS1 EQ digests, suggesting additional cleavage sites not identified by semi-tryptic peptides. As with ADAMTS4, cleavage at S^77^-V^78^ was identified by 6 semi-tryptic peptides exclusively in WT ADAMTS1 digests, with the highest peptide abundance of any significant peptide, and the cleavage site was supported by 2 tryptic peptide singletons in the EQ digests (residues 54–79 and 58–79, Table 2, Fig. 2C, D). The adjacent cleavage site, Q^76^-S^77^, was similarly identified by 6 semi-tryptic peptides and were among the most abundant peptides, and 2 tryptic peptides exclusively in the WT and EQ digests, respectively. We also found strong evidence for cleavage at D^248^-G^249^ in EGF4 (1 semi-tryptic peptide with higher abundance and 2 tryptic peptides with lower abundance) and A^639^-V^640^ (1 semi-tryptic with higher abundance and 1 tryptic peptide with lower abundance) in the CTD. We detected 3 tryptic COMP peptides with significant *z*-scores in the WT ADAMTS1 digests, which could arise from a trypsin-like activity of ADAMTS1 and 200 semi-tryptic COMP peptides with significant *z*-scores in the ADAMTS1 EQ control digests whose origins are unclear (Fig. 2C). The cleavage sites shared by ADAMTS1 and ADAMTS4 were Q^76^-S^77^ and S^77^-V^78^ in the NTD, and Q^753^-L^754^ in the CTD (Fig. 2E).

We further investigated if semi-tryptic peptides generated by ADAMTS1 or ADAMTS4 activity were present in the degradome of cartilage or synovial fluid of OA patients [55–57] and identified several proteolytic peptides that were also detected in the patient samples (Table 3).

### ADAMTS4 cleaves COMP efficiently at the S^77^-V^78^ bond

We further investigated the prominent S^77^-V^78^ cleavage site located in the C-terminal linker region of the NTD, since this proteolytic event could disrupt the pentamerization of COMP and be potentially responsible for the ~70 kDa band detected by the polyclonal anti-COMP antibody in the presence of both ADAMTS1 and 4 (Fig. 1H–J). Importantly, one neoepitope originating from cleavage at this site (QQS^77^) was previously detected in the synovial fluid of patients with knee pain, OA, and rheumatoid arthritis using LC-MS/MS and neoepitope antibodies [56,58] as well as in our recent degradomic analyses of OA cartilage [55] (Table 3) and is thus clinically relevant to joint disease. We incubated hCOMP in the presence of ADAMTS4, ADAMTS4 EQ or buffer. The reaction products were run on SDS-PAGE under non-reducing conditions and immunoblotted with anti-QQS^77^ neoepitope antibody (Fig. 2F). In the presence of ADAMTS4, but not ADAMTS4 EQ or the buffer control, 4 major COMP cleavage fragments containing the QQS^77^ neoepitope were detected. Their sizes at >250, ~200, >100 and <50 kDa corresponded to the multimeric N-terminal cleavage fragments generated by 2, 3, 4, or 5 cleavages in pentameric COMP. The band of ~50 kDa, therefore, represents the fully cleaved NTD pentamer. The released cleaved monomers that make up the C-terminal cleavage fragments were not visible here as they did not contain QQS^77^ neo-epitope. Bands of lower molecular weight were also detected with the QQS^77^ neo-epitope antibody and could indicate further proteolysis within the NTD of COMP.

Among the 31 other putative ADAMTS4 cleavage sites in COMP (Fig. 2E), we additionally assessed cleavage at the F^577^-N^578^ bond in the CTD (Table 1), since COMP peptides resulting from cleavages at this site were previously identified in the synovial fluid of patients with OA, acute trauma, reactive arthritis and rheumatoid arthritis (RA) [56,58] (Table 3). However, antibodies directed against the N- or C-terminal neoepitope resulting from cleavage at the site (*i.e*. anti-TAF^577^ or anti-^578^NGV) failed to detect the expected bands in ADAMTS4 digests of COMP under conditions where the QQS^77^ neoepitope was abundant (data not shown). We also analyzed the ability of other metalloproteinases to cleave at the S^77^-V^78^ bond. Compared to ADAMTS4, only ADAMTS1 and ADAMTS5 showed a very weak activity at this site after 24 h incubation, whereas neither ADAMTS7 nor ADAMTS8 showed any proteolytic activity, even after 24 h incubation (Fig. 2G). The presence of 2 mM ZnCl_2_ did not affect the ability of ADAMTS7 to cleave at this site (Supplementary Fig. 1B). Additionally, MMP1, MMP3 and MMP7 did not cleave COMP at the S^77^-V^78^ cleavage site under these conditions (Supplementary Fig. 5).

Taken together, the data demonstrates that ADAMTS4 cleaves COMP at the S^77^-V^78^ bond in the NTD to generate neopeptides previously detected in human OA samples.

### ADAMTS4 COMPase activity requires its spacer domain

**Fig. 3.**
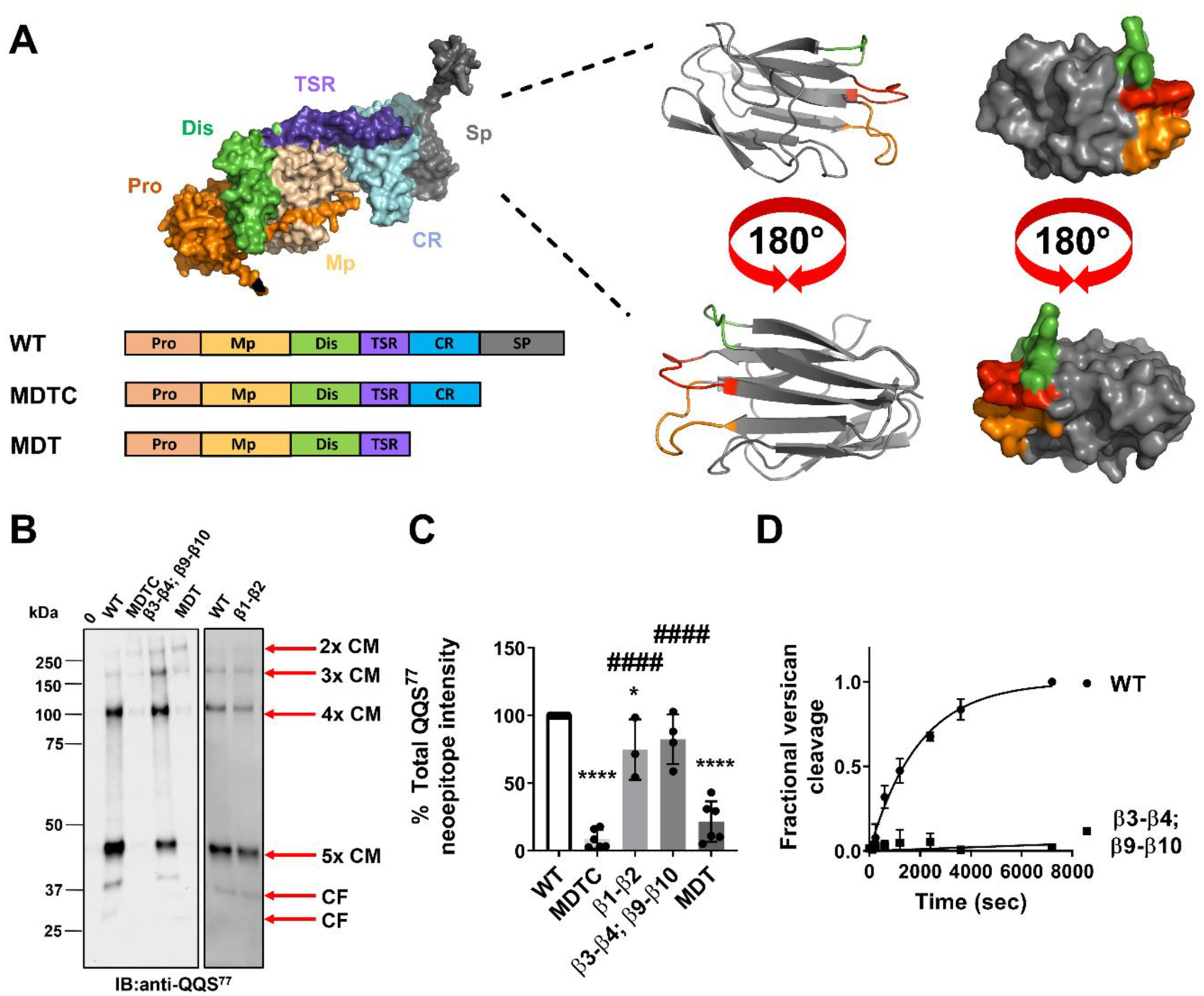
Determination of structural requirements for ADAMTS4 COMPase activity. **A)** AlphaFold model of ADAMTS4 (AlphaFold ID AF-O75173-F1, visualized using PyMOL, version 2.5.4) and schematic of ADAMTS4 domain deletion variants (signal peptide and the C-terminal FLAG tag are not shown). The insert shows AlphaFold model of the Sp domain (left as a cartoon, right as a surface) highlighting the loops previously shown to be involved in versicanase activity: β1-β2 (residues 693–698, green), β3-β4 (residues 717–724, orange), and β9-β10 (residues 788–795, red). **B)** ADAMTS4 Sp is required for efficiently cleaving COMP at the S^77^-V^78^ bond. hCOMP (520 nM) was incubated with ADAMTS4 WT, MDTC, β3-β4; β9-β10, MDT, β1-β2, (each at 12 nM) or buffer alone (0) for 2 h at 37 °C. Samples were then subjected to SDS-PAGE under non-reducing conditions and probed with a neoepitope anti-QQS^77^ polyclonal anti-COMP antibody. The vertical line indicates where lanes were digitally rearranged from original blots for clarity of presentation. Blot representative of three independent experiments. CF, cleavage fragment; CM, cleaved monomer; IB, immunoblot. **C)** Densitometric analysis of COMP cleavage at the S^77^-V^78^ site. All the anti-QQS^77^-reactive bands were quantified and the signal in the presence of WT enzyme was set as 100 %. The data are presented as average ± SD (*n* = 3–6). Statistical analysis was performed using one-way ANOVA with Tukey correction. *, *p* < 0.05 compared to WT; ****, *p* < 0.0001 compared to WT; ####, *p* < 0.0001 compared to MDTC. **D)** Time course experiments for cleavage of 50 nM versican V1–5GAG by ADAMTS4 and β3-β4; β9-β10 (each at 1 nM). Data are presented as mean ± SD (*n* = 3). The solid lines represent a non-linear regression fit of the data as described in the Experimental Procedures.

The currently known substrate repertoire of ADAMTS4 predominantly comprises proteoglycans *i.e*., aggrecan, versican, decorin, and biglycan [59,60]. We therefore tested whether the structural requirements for ADAMTS4 proteolytic activity were similar for proteoglycans and COMP. Generally, ADAMTS proteases use their non-catalytic ancillary domains to bind their substrates with high affinity and enhance the rate of proteolysis [60–64]. For instance, ADAMTS4 requires both the Sp and CR domains to bind and cleave aggrecan [63] and versican [60]. To identify which domains of ADAMTS4 are important for COMP cleavage at the S^77^-V^78^ cleavage site, two truncated variants were expressed and purified [59,60,63], one lacking the Sp domain (MDTC), and the other one lacking both the Sp and CR domains (MDT) (Fig. 3A). Their concentration was determined by active-site titration with tissue inhibitor of metalloproteinase 3 (TIMP3) using a quenched-fluorescent peptide (QF) as a substrate [60] and their COMPase activity was compared with that of WT enzyme (Fig. 3B). Densitometric analysis of all anti-QQS^77^ reactive bands revealed that the activity of MDTC was significantly reduced (~90 %) compared with that of WT ADAMTS4 (Fig. 3C). Deletion of the CR domain (variant MDT) did not result in further significant reduction in COMPase activity compared to MDTC. These data suggest that the Sp is a key determinant of COMPase activity, but due to the low activity of MDTC we cannot exclude additional contributions from the CR domain.

We have previously shown [60] that ADAMTS4 mainly uses two loops in the Sp domain to bind versican (β3-β4 and β9-β10, corresponding to residues 717–724 and 788–795, respectively) (Fig. 3A). To test if the same loops were involved in binding COMP, we generated a new variant where both the β3-β4 and β9-β10 loops were replaced with those of a distantly related protease, ADAMTS13, which is unable to cleave proteoglycans [65]. This variant (β3-β4; β9-β10) was expressed, purified, and quantified exactly like the other ADAMTS4 variants (Supplementary Fig. 6). Kinetic assays confirmed that β3-β4; β9-β10 had very weak versicanase activity compared to WT ADAMTS4 (*k*_cat_/*K*_m_ values: WT, 2.4 ± 0.36 × 10^5^; β3-β4; β9-β10: ~0.001 × 10^5^; >2400-fold reduction) (Fig. 3D). When tested for its ability to cleave COMP at the S^77^-V^78^ cleavage site, β3-β4; β9-β10 showed a modest (~20 %) reduction in COMPase activity, although this was still significantly higher than that of MDTC, lacking the entire Sp (Fig. 3B and C). These results prompted us to test an additional variant, namely β1-β2, where residues 693–698, adjacent to the β9-β10 loop (Fig. 3A) were swapped with those of ADAMTS13 and whose versicanase activity was reduced 2-fold compared to WT ADAMTS4 [60]. β1-β2 exhibited a modest (25 %) but significant reduction in COMPase activity compared to WT ADAMTS4 (Fig. 3B and 3C).

These results show that the Sp is required for ADAMTS4 COMPase activity and that the β1-β2 loop is involved in the interaction.

### Molecular modelling predicts S^77^-V^78^ as a favored site for ADAMTS4 cleavage in the COMP N-terminal domain

**Fig. 4.**
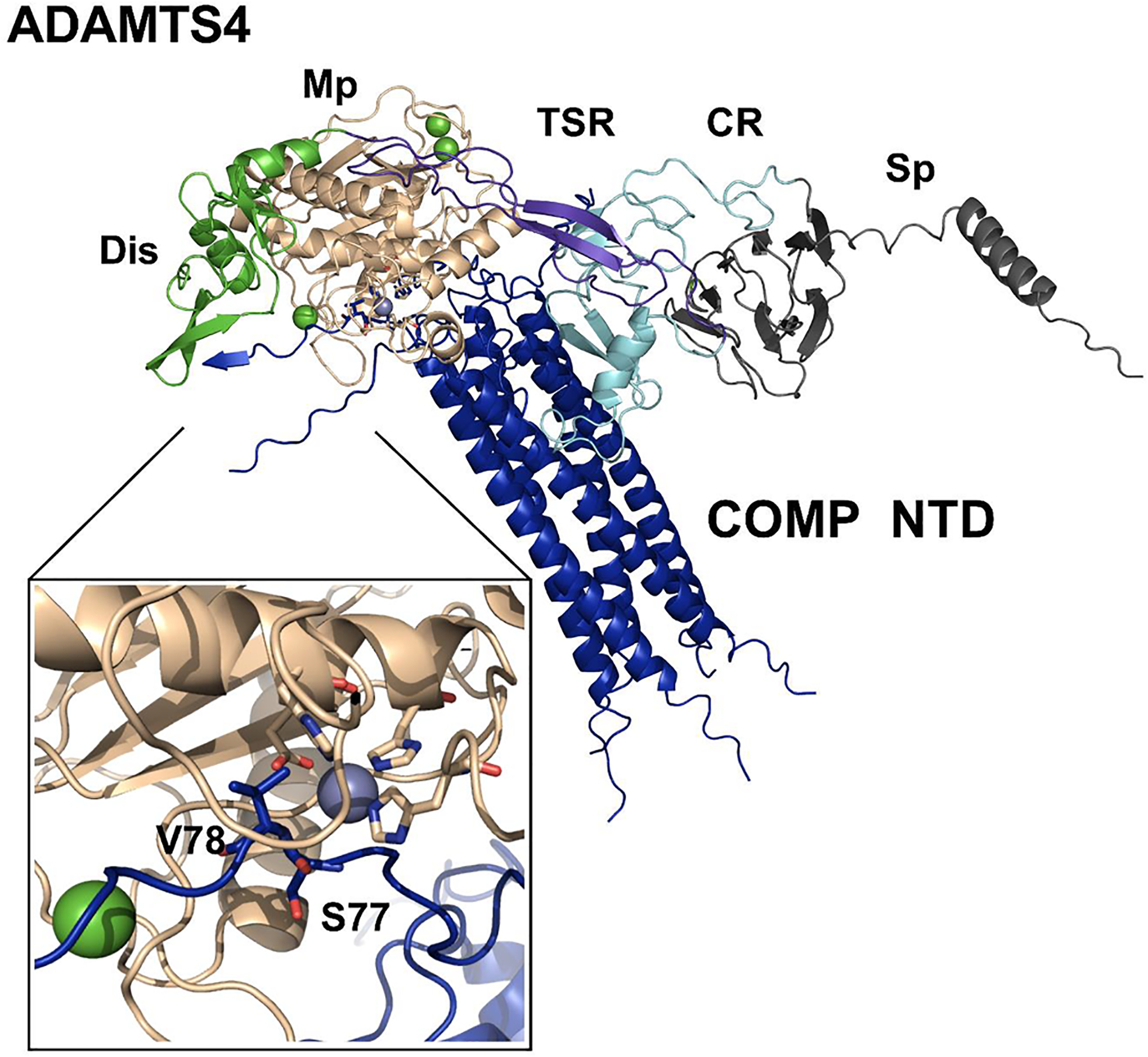
AlphaFold-Multimer prediction of the interaction between mature ADAMTS4 and the pentameric N-terminal region G^22^-R^86^ of COMP pentamer. Shown is the best ranked prediction generated with the ColabFold pipeline. The model correctly predicts the COMP NTD to form a pentamer and the S^77^-V^78^ site of one of the 5 chains to lie in the active site of ADAMTS4. The domains of ADAMTS4 are shown in wheat (Mp), green (Dis), purple blue (TSR), cyan, (CR) and gray (Sp). The COMP NTD is shown in blue. In the close-up inset of the active site, the S^77^-V^78^ residues of COMP, the three ADAMTS4 histidine residues coordinating the zinc ion (H361, H365, and H371) and the catalytic glutamic acid (E362) are shown as sticks with oxygen and nitrogen atoms in red and blue, respectively. In ADAMTS4, the catalytic zinc and calcium ions are shown as grey and green spheres, respectively.

We used AlphaFold-Multimer [66,67] to model the molecular interactions between ADAMTS4 and COMP. The input sequences provided to AlphaFold-Multimer were 5 chains of the COMP N-terminal sequence G^22^-R^86^ and mature (*i.e*. with the prodomain-removed) ADAMTS4 sequence (R^218^-K^837^). AlphaFold-Multimer produced 5 models. In all five models, AlphaFold accurately predicted multimerization of COMP (Fig. 4), matching the 3D pentameric structure of the NTD determined by X-ray crystallography [68] and rotary shadowing electron microscopy of the whole pentameric protein [46]. Interestingly, in the highest ranked model, the S^77^-V^78^ site of one of the 5 chains is predicted to lie in the active site, positioning this scissile bond on top of the catalytic zinc and E^362^ which coordinates the water molecule that drives hydrolysis. We were unable to include full-length COMP in the model and therefore could not model any interactions involving the ADAMTS4 CR and Sp domains with other COMP domains (Fig. 4).

### Selective ADAMTS4 inhibition decreases generation of the QQS^77^ neoepitope in human OA cartilage explants

**Fig. 5.**
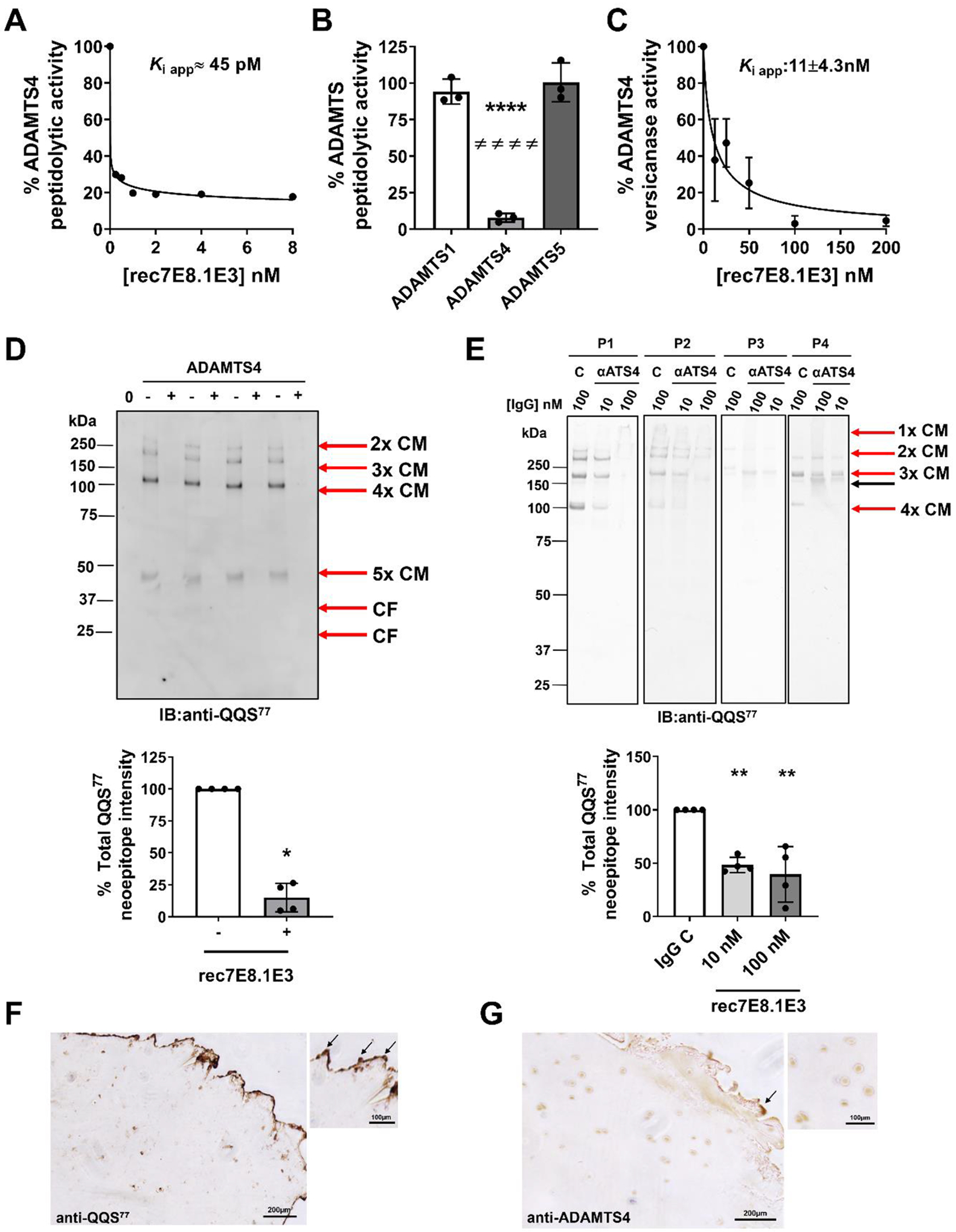
Selective inhibition of ADAMTS4 significantly decreases QQS^77^ neoepitope generation in human OA cartilage explants. **A)** Selectivity profile of rec7E8.1E3. ADAMTS1 (10 nM), ADAMTS4 (3 nM) and ADAMTS5 (10 nM) were incubated with purified rec7E8.1E3 (10 nM) for 1 h at 37 °C before addition of the QF substrate Fam-AE~LQGRPISIAK-TAMRA (40 μM, ADAMTS1 and ADAMTS4) or Fam-TESESRGAIYKK-TAMRA (40 μM, ADAMTS5). The data are presented as average ± SD (*n* = 3). Statistical analysis was performed using one-way ANOVA with Tukey correction. ****, *p* < 0.0001 compared to ADAMTS1; ####, *p* < 0.0001 compared to ADAMTS5. **B)** Inhibition of ADAMTS4 peptidolytic activity by rec7E8.1E3 using a QF peptide substrate. ADAMTS4 (0.2 nM) was incubated with increasing concentrations of purified rec7E8.1E3 (0–8 nM) for 1 h at 37 °C before addition of the QF substrate Fam-AELQGRPISIAK-TAMRA (3.5 μM). Each point represents the average of two technical replicates. Data were fitted to the Morrison equation. **C)** Inhibition of ADAMTS4 versicanase activity by rec7E8.1E3. ADAMTS4 (5.5 nM) was incubated with increasing concentrations of purified rec7E8.1E3 (0–200 nM) for 1 h at 37 °C before addition of a truncated versican substrate (V1–5GAG, 50 nM). Samples were then subjected to versikine ELISA. The data are presented as average ± SD (*n* = 3). **D)** Inhibition of ADAMTS4 COMPase activity at the S^77^-V^78^ site. hCOMP (520 nM) was incubated with ADAMTS4 (12 nM) or buffer alone (0) for 2 h at 37 °C either in the presence (+) or absence (−) of rec7E8.1E3 (240 nM). Samples were then subjected to SDS-PAGE under non-reducing conditions and probed with a neoepitope anti-QQS^77^ polyclonal anti-COMP antibody. Top panel: immunoblot for 4 independent replicates. CF, cleavage fragment; CM, cleaved monomer; IB, immunoblot. Bottom panel: densitometric analysis of the immunoblot. The anti-QQS^77^-reactive bands were quantified and the signal in the absence of rec7E8.1E3 was set as 100 %. The data are presented as average ± SD (*n* = 4). *, *p* < 0.05 by Mann-Whitney test. **E)** Effect of ADAMTS4 inhibition on COMP degradation in human knee OA cartilage explants (*n* = 4, P1-P4). After 24 h incubation with 7E8.1E3 (αATS4, 10 and 100 nM) or isotype control (C, 100 nM), the medium was collected, precipitated with trichloroacetic acid, dissolved in SDS sample buffer and subjected to SDS-PAGE under non-reducing conditions followed by immunoblotting with anti-QQS^77^ neoepitope antibody. Top panel: immunoblot for 4 patients. The black arrow indicates a non-specific band detected by the secondary antibody and matching the size of immunoglobulins (IgG). The vertical lines indicate where lanes were digitally rearranged from original blots for clarity of presentation. CM, cleaved monomer; IB, immunoblot. Bottom panel: densitometric analysis. The anti-QQS^77^-reactive bands were quantified and the signal in the presence of the isotype control (IgG C) was set as 100 %. The data are presented as average ± SD (*n* = 4). Statistical analysis was performed using one-way ANOVA with Tukey correction. **, *p* < 0.01 compared to isotype control. **F, G)** Representative immunostaining (*n* = 2) of advanced human OA articular cartilage showing COMP QQS^77^ **(F)** and ADAMTS4 **(G)** localization (brown). Arrows indicate intense staining at the superficial zone.

Based on the ability of ADAMTS4 to efficiently cleave COMP at the S^77^-V^78^ bond, we hypothesized that ADAMTS4 was responsible for generation of the clinically relevant QQS^77^ neoepitope that has previously been detected in the synovial fluid of OA patients [56,58]. We tested this by selectively inhibiting ADAMTS4 proteolytic activity in human OA cartilage explants. While no small molecule inhibitor has been reported to be so far entirely selective for ADAMTS4 [69], researchers at GlaxoSmithKline developed a monoclonal antibody, 7E8.1E3, that inhibits ADAMTS4 while sparing ADAMTS1, ADAMTS5, ADAMTS13 and a panel of MMPs including MMP1, 3, 9 and 13 [70]. 7E8.1E3 recognizes epitopes in the Mp/Dis domains of ADAMTS4 and was effective in inhibiting aggrecan cleavage in human OA cartilage explants stimulated with IL-1β/oncostatin M [70]. 7E8.13 therefore provided a valuable tool to test the impact of ADAMTS4 inhibition on COMP degradation in human OA cartilage samples. To generate recombinant 7E8.1E3, the complementarity determining regions of the original antibody were engrafted into a mouse IgG1 scaffold and cloned onto separate plasmids for the heavy and light chain, respectively. A 6x His tag and a 3x/FLAG tag were attached at the C-terminus of the heavy chain constructs. The two plasmids were transiently transfected into HEK293T cells and the secreted antibody was purified using immobilized metal affinity chromatography (Supplementary Fig. 7). When tested in a QF peptide cleavage assay, this recombinant 7E8.1E3 (rec7E8.1E3) potently inhibited ADAMTS4, with an estimated *K*_i_ value of ~45 pM, very close to the *K*_i_ app value of the parent antibody (35 pM), as measured using bovine aggrecan as a substrate [70] (Fig. 5A). Of note, tight inhibition coupled with the low turnover number/sensitivity of the QF peptide currently used to monitor ADAMTS4 activity [71] did not allow us to obtain an accurate estimate of the *K*_i_ value. We confirmed the selectivity of rec7E8.1E3 for ADAMTS4 against ADAMTS1 and ADAMTS5 using QF peptide substrates (Fig. 5B). Rec7E8.1E3 inhibited ADAMTS4 versicanase activity with a *K*_i_ app value of 11 nM (Fig. 5C) and was able to completely inhibit ADAMTS4 COMPase activity at the S^77^-V^78^ site (Fig. 5D).

After having assessed that rec7E8.1E3 retained the inhibitory potency and selectivity of the parent antibody, we tested its ability to inhibit the generation of neoepitope QQS^77^ in human knee OA cartilage explants. The explants were incubated either in the presence of rec7E8.1E3 (10 or 100 nM) or control IgG1 (100 nM) for 24 h and the medium was subjected to non-reducing SDS-PAGE and immunoblotting with anti-total COMP or the QQS^77^ neoepitope antibodies (Fig. 5E). Robust QQS^77^ neoepitope generation was observed in the presence of control IgG1. Although highly variable between different patients, the immunostaining pattern closely resembled that observed with recombinant hCOMP (Fig. 2F and 2G). Specific anti-QQS^77^ reactive bands were significantly decreased by both concentrations of rec7E8.1E3 as compared to IgG1 control (Fig. 5E). No effect of rec7E8.1E3 on full length COMP levels was observed (Supplementary Fig. 8). These results suggest a major role of ADAMTS4 in generating QQS^77^ neopeptides in human OA cartilage.

For localization in OA cartilage, we performed immunohistochemical staining with QQS^77^ neoepitope antibody and ADAMTS4. The QQS^77^ neoepitope immunosignal was predominately localized to the fibrillated surface area, indicating accumulation in damaged cartilage (Fig. 5F). The QQS^77^ neoepitope immunosignal was also localized to and around chondrocytes. ADAMTS4 immunosignal was detected in the damaged superficial area and around chondrocytes (Fig. 5G), suggesting its colocalization with the cleaved COMP.

## Discussion

The present results indicate that selective inhibition of ADAMTS4 is sufficient to significantly decrease QQS^77^ neoepitope generation in human OA cartilage, and together with the observed high efficiency cleavage at the S^77^-V^78^ site, suggests that ADAMTS4 may be responsible for generating the majority of QQS^77^ neoepitope in OA patients. Further study is warranted to investigate this in a larger cohort of patients.

Like many structural ECM components, COMP levels are proteolytically regulated to preserve cartilage integrity as part of a physiological remodeling process [41]. The responsible proteases are thought to belong to the MMP and ADAMTS families of metalloproteinases [41]. Upregulated metalloproteinase activity leads to COMP degradation, a hallmark of degenerative joint diseases such as OA [25,41]. Using purified COMP and proteases, in 1998 Ganu et al. identified COMP as a substrate for MMP1, 3, 13, and 9 [72]. Although at that time the identity of the resulting COMP fragments was not elucidated, their size was similar to those detected in synovial fluid from OA and RA patients [56]. However, given the high enzyme concentrations (268 nM, enzyme/monomeric COMP ratio: 0.5) and incubation times (3–20 h) [72] used in that study, it is questionable whether COMP represents a physiological substrate for these MMPs. In fact, when we used lower enzyme concentrations (enzyme/monomeric substrate ratio 1:0.004) we did not detect any COMPase activity by MMP1 and MMP3 (Fig. 1J). The majority of COMP degradation in bovine nasal cartilage and human menisci stimulated with pro-inflammatory cytokines occurs within the first two weeks of culture [42,73], when MMP activity is undetectable [42,74], supporting the notion that MMPs are not major COMPases *in vivo*. Within this window, most of the aggrecan is known to be depleted from the ECM [74], a proteolytic activity attributed to ADAMTS proteases, in particular ADAMTS4 and ADAMTS5 [45]. In 2003, Dickinson et al. reported that purified ADAMTS4 cleaved COMP, generating a fragment with molecular weight of 110 kDa (fragment-110) under reducing conditions, while no cleavage by ADAMTS1 and 5 was detected [42]. Fragment-110 resulted from an N-terminal cleavage event and migrated between 67 and 80 kDa under non-reducing conditions [42,72], similar in size to the 70 kDa fragment that we detected with a polyclonal anti-COMP antibody when both iCOMP (Fig. 1E) and hCOMP (Fig. 1H–J) were incubated with ADAMTS1, 4, and 5. We confirmed that ADAMTS4 was the most potent COMPase, followed by ADAMTS1, while ADAMTS5 COMPase activity was detected only upon prolonged incubation (24 h). Additionally, we extended these findings to ADAMTS7 and ADAMTS8. ADAMTS8 is phylogenetically closer than ADAMTS7 to ADAMTS1, 4, and 5 but while these three proteases act as proteoglycanases, ADAMTS8 did not show any detectable activity against versican, aggrecan or biglycan *in vitro* [75]. The lack of both proteoglycanase and COMPase activity suggests a potentially distinct substrate repertoire for ADAMTS8 compared to ADAMTS1, 4 or 5. ADAMTS7 was previously reported to cleave COMP, generating one fragment of 100 kDa and two fragments migrating between 95 and 51 kDa under non-reducing conditions [43]. In contrast, we were unable to detect any COMPase activity by ADAMTS7 (12nM, 24 h) when both iCOMP (Fig. 1E) and hCOMP (Fig. 1H and 1I) were used as substrates. The ADAMTS7 construct we used for these experiments retained proteolytic activity against a number of substrates, such as latent TGF-β–binding protein (LTBP)4 [48]. Since the ADAMTS7 construct was truncated before the C-terminal PLAC domain, it retained the C-terminal TSRs that are supposedly required for ADAMTS7 interaction with COMP and, consequently, for full COMPase activity [43]. At present, we cannot provide an explanation for the discrepancy with previous reports of COMP cleavage by ADAMTS7. Two independent, unbiased, substrate identification studies for ADAMTS7 using N-Terminal Amine Isotopic Labeling of Substrates (N-TAILS) identified several ADAMTS7 substrates, which did not include COMP [48,76].

Using our recently described *z*-score method [54], we mapped the cleavage sites in COMP for both ADAMTS4 and ADAMTS1, identifying 32 and 17 cleavages by ADAMTS4 and ADAMTS1, respectively, of which only 3 were shared by both proteases. The inclusion of these newly identified cleavage sites in a list of cleavage sites generated by ADAMTS4 on 17 ECM substrates (Supplementary Table 1) confirmed the preference for a negatively charged amino acid such as glutamic acid or aspartic acid at the P1 position, while the P1′ pocket has a broader specificity (Supplementary Fig. 9), most likely due to the relatively large size of the S1′ pocket [77]. Arginine and glycine were over-represented in P2′ and P3′ positions, respectively, while glutamine was favored in P2. This analysis agreed well with a consensus motif previously generated by incubating ADAMTS4 with a 13-mer peptide library [78]. Due to the limited number of substrates currently reported for ADAMTS1 in the MEROPS database [79], we were not able to generate IceLogo plots for this protease.

By digesting human articular cartilage with recombinant ADAMTS4, Zhen et al. identified D^530^-F^531^ as an additional cleavage site in COMP [39]. In our *in vitro* analysis, this cleavage site was identified only in the ADAMTS1 digest. While we used full-length ADAMTS4 in our experiments, Zhen et al. used a form truncated after the TSR (residues 213–579). This truncated construct does not contain the C-terminal CR and Sp domains which we have shown here to be essential for ADAMTS4 COMPase activity (Fig. 3), suggesting that truncated ADAMTS4 may have a different cleavage site specificity compared to the full-length enzyme.

Some of the ADAMTS4 cleavage sites we identified in COMP were affected by mutations in patients with skeletal dysplasia: D^310^-A^311^ (TSP2) (D310 V) [80], M^717–^R^718^ (CTD) (R718P, [80], and R718W [81]). The D310 V and R718P COMP mutations may decrease susceptibility to ADAMTS4 proteolysis based on the Icelogo plots (Supplementary Fig. 9), although this should be demonstrated experimentally using mutated recombinant COMP. Whether disease-causing *COMP* mutations are indeed related to dysregulated ADAMTS4-mediated cleavage warrants further investigation. Another point deserving additional studies is whether distinct glycosylation patterns on COMP may affect proteolytic susceptibility. Zaia et al. [82] showed that COMP purified from human cartilage contains 2 *N*-linked glycans but no *O*-linked glycans. *N*-linked glycans are known to affect rate of proteolysis at peptide bonds in the vicinity of their attachment sites [83]. The *N*-linked glycosylation site at N^121^ (N^101^ in mature COMP) is the glycosylation site nearest to the important S^77^-V^78^ cleavage site. This is probably too far from S^77^-V^78^ to affect proteolysis, but molecular dynamics studies could give more insight in how this glycan may affect the folding and susceptibility to proteolysis. The exact nature of the glycan at this site and potential alterations in disease and aging should be established by mass spectrometry. Importantly for this study, HEK293T cells express all the enzymes needed for full mammalian *N*-linked glycosylation. Our experiments using COMP expressed in insect cells, which contain much shorter *N*-linked glycans, did not differ from those with HEK293T cell-expressed COMP, suggesting *N*-linked glycosylation does not majorly affect proteolysis by the ADAMTSs and MMPs tested.

The cleavage sites we identified *in vitro* by semi-tryptic peptide analysis are further rendered translationally relevant by their occurrence in human OA cartilage and synovial fluid (Table 3). We initially focused our investigation on the S^77^-V^78^ cleavage site since peptides generated by both ADAMTS1 and ADAMTS4 activity were previously identified in the synovial fluid of patients with acute trauma, OA, and RA [56]. While no studies have assessed the association between QQS^77^ neopeptides and OA progression, the molar ratio of COMP QQS^77^ neopeptide/total COMP was significantly associated with RA progression [58]. Here and in previous work [56,58,73], using a neoepitope antibody, we have shown that cleavage at S^77^-V^78^ in the NTD disrupts the pentameric organization of COMP and may be potentially detrimental to cartilage integrity.

The QQS^77^ neoepitope-containing cleavage fragments detected here were previously identified in human cartilage explants treated with tumor necrosis factor-α and interleukin (IL)–6/soluble IL-6 receptor [56], which also upregulate ADAMTS4 [84]. Based on the kinetics of the release of the QQS^77^ neoepitope in cartilage explant studies [56,73,85], it is most likely that COMP is degraded in cartilage before release into synovial fluid.

We have shown that ADAMTS4 inhibition significantly decreased generation of the QQS^77^ neoepitope in cultured human OA cartilage explants by >50 %, suggesting that in this system ADAMTS4 is primarily responsible for cleaving COMP at S^77^-V^78^, while residual activity at this site may be due to other proteases such as ADAMTS1 We did not detect significant inhibition of total COMP degradation in the presence of the anti-ADAMTS4 inhibitory antibody (Supplementary Fig. 8), indicating that inhibition of ADAMTS4 alone is not sufficient to block COMP degradation and that other proteases cleaving at other bonds are involved. Indeed, a recent N-TAILS analysis identified COMP as a substrate of the serine protease HtrA1 [55]. Susceptibility to proteases other than MMPs and ADAMTSs is further corroborated by the finding that addition of broad-spectrum metalloproteinase inhibitors only partially inhibited COMP degradation in bovine nasal cartilage explants treated with IL-1α [42]. Therefore, we can conclude that proteases belonging to several families cooperate to degrade COMP [41] and that therapeutic inhibition of ADAMTS4 for OA therapy would likely have a modest effect on COMP protection, but the biological impact could depend on specific bioactivities possibly residing in distinct COMP fragments. This is another area of research that deserves further investigation.

*Ex vivo* studies using human knee cartilage treated with cytokines showed that cleavage of proteoglycans preceded that of COMP at the S^77^-V^78^ site since approximately 30–50 % of the glycosaminoglycan pool was released into the medium before detection of the QQS^77^ neoepitope [56]. Taken together, this data suggests that ADAMTS proteoglycanases such as ADAMTS1 and ADAMTS4 preferentially cleave proteoglycans such as aggrecan over COMP, either because of their different affinity for the substrate or a different availability of COMP to proteolytic attack at the early stage of ECM degradation. Although we have shown in this study that ADAMTS4 requires the Sp domain for efficiently cleaving COMP and previous studies indicated that the Sp domain is similarly required for efficient cleavage of proteoglycans [60,62], the reduction in COMPase activity of the Sp loop variants is less dramatic than that in versicanase activity (Fig. 3C and 3D), suggesting relative differences in their affinity and exosite engagement for COMP and versican, respectively. Future *in vitro* kinetic studies on COMP cleavage by ADAMTS4 will allow us to compare the specificity constants for COMP with that of versican [60].

The relevance of our findings may not be limited to OA, as COMP has been shown to play a role in the progression of several types of cancer, pulmonary arterial hypertension, idiopathic pulmonary fibrosis, and skin fibrosis (reviewed in [86]), all diseases where circulating levels of the QQS^77^ neoepitope have not been measured in comparison to control subjects.

To summarize, we have shown that ADAMTS4 is a potent COMPase with the distinct ability to cleave COMP at the S^77^-V^78^ bond, generating neopeptides also found in human OA cartilage and synovial fluid. By cleaving both COMP and aggrecan, ADAMTS4 therefore is likely to play a crucial role in modulating structural integrity of the cartilage ECM. Future assessment of ADAMTS4 inhibitors as disease-modifying OA drugs could leverage these findings and employ simultaneous monitoring of COMP and aggrecan degradation as readouts of ADAMTS4 activity and drug response.

## Experimental procedures

### Protein expression and purification

#### Proteases and proteoglycans.

The constructs coding for human full-length ADAMTS1, 4, 5, and 8 with a C-terminal FLAG tag (DYKDDDDK) were described previously [60,75]. ADAMTS4 variants were generated using site-directed mutagenesis and confirmed through sequencing. The versican V1–5GAG plasmid, comprising amino acids 21–694 of V1 with C-terminal c-myc and 6x His tags, was described previously [60]. Expression and purification of ADAMTS variants and V1–5GAG were performed as previously reported [60]. The ADAMTS7 variant used in this study (ADAMTS7-T8) is truncated before the C-terminal PLAC domain [48]. It is fused at the C-terminus to a myc and 6xHis tag and was expressed and purified as previously described [48]. ADAMTS concentrations were determined by active-site titration with TIMP3 (ADAMTS1, ADAMTS4 and ADAMTS5) [60,64] or TIMP4 (ADAMTS7) [48]. For determination of ADAMTS8 concentration, optical density was used [75]. Full-length MMP1 and MMP3 were purified to homogeneity as before [87,88] and full-length MMP7 was purchased from R&D (Cat. number: 907-MP-010).

#### hCOMP.

Expression and purification of recombinant full-length human hCOMP with a C-terminal 6x His tag were previously described [51]. Briefly, human Freestyle 293-F cells (Life Technologies, Waltham, MA, USA) were transfected with the mammalian episomal expression plasmid pCEP4-BM40-hisEK [89], containing cDNA for COMP. COMP protein was purified from conditioned medium by sequential affinity (HisTrap Excel), ion exchange (Mono Q) and size exclusion (Superdex S200 Increase) chromatography. The total purified protein yield was 0.24 mg/L culture medium.

#### iCOMP expression and purification.

To enable COMP expression in the *Drosophila melanogaster* Schneider cells 2 (S2) cell line, human COMP cDNA was obtained from Source BioScience, amplified by PCR and cloned into the S2 expression vector pMT-BiP-PURO using the Gibson assembly^®^ Master Mix (New England Biolabs) following manufacturer’s instructions. The vector fuses the Drosophila BiP secretion signal to Gly^22^ of COMP to enable entry into the S2 cell secretory pathway. The BiP signal peptide is cleaved off prior to secretion by the cells to provide a native N terminus. C-terminal V5 and 6x His tags were added to facilitate detection and purification. Recombinant expression in the S2 expression system was performed as described previously for ADAMTS13 [90]. iCOMP was purified using a Ni^2+−^Sepharose column (GE Healthcare) previously equilibrated with 3 column volumes TBS (20 mM Tris-HCl, pH 7.4, 150 mM NaCl). Following binding, the column was washed with TBS containing 10 mM imidazole and bound proteins were eluted using a linear gradient (10–300 mM) of imidazole. Eluted fractions containing COMP were subjected to SDS-PAGE, pooled, concentrated on Amicon Ultra spin columns (10 kDa cut-off) and dialyzed extensively against TBS. Pentameric COMP concentration was measured using optical density and calculated according to the Beer–Lambert law using extinction coefficients 370,950 *M*^−1^ cm^−1^ on the Expasy ProtParam web tool.

#### Anti-ADAMTS4 antibody.

To generate the anti-ADAMTS4 monoclonal antibody, the sequences coding for 7E8.1E3 complementarity determining regions [70] were custom- synthesized by Invitrogen, introduced into a mouse IgG1 scaffold and cloned into separate plasmids in pcDNA3.1^+^ encoding heavy and light chain using *KpnI*/*NotI* restriction sites. To facilitate detection, a 6x His tag and a 3x FLAG tag were attached at the C-terminus of the heavy chain. For antibody expression, human embryonic kidney cells expressing the SV40 large T antigen (HEK293T) were cultured in Minimum Essential Medium Eagle (MEM) (Sigma-Aldrich, Gillingham, UK) with 10 % fetal bovine serum (FBS, Labtech), 1 U/mL Penicillin and 0.1 mg/mL Streptomycin (Pen/Strep) (Sigma-Aldrich), 2 mM L-glutamine (Life Technologies) and 1X non-essential amino acids (Sigma-Aldrich) at 37 °C, with 5 % CO_2_. After reaching >75 % confluence, cells were washed with phosphate-buffered saline to remove FBS and the medium was replaced with Opti-MEM Gibco^™^ (Life Technologies) containing Pen/Strep (1 U/ml and 0.1 mg/mL, respectively) and 2 mM CaCl_2_. The plasmids coding for the antibody heavy and light chain were transiently co-transfected into HEK293T cells at 1:1 ratio using polyethylenimine (PEI MAX) (Polysciences Europe GmbH, Germany) (PEI/cDNA ratio: 3.6). Following transfection, cells were incubated for 3 days before harvesting. The conditioned medium was harvested and centrifuged for 20 mins at 1500 × *g*, followed by filtration (0.45 μm) to remove cell debris and concentrated (10-fold) on a tangential flow filtration system (Millipore) with a 10 kDa cut-off. The secreted antibody was purified using a Ni^2+−^Sepharose column (GE Healthcare) previously equilibrated with 3 column volumes of HBS (20 mM Hepes, pH 7.4, 150 mM NaCl) on an ÄKTA^™^ start protein purification system (Cytiva, Little Chalfont, UK). Following binding, the column was washed with HBS containing 10 mM imidazole and bound proteins were eluted using a linear gradient (10–300 mM) of imidazole. Eluted fractions containing the full-length antibody were subjected to SDS-PAGE, pooled, concentrated on Amicon Ultra spin columns (10 kDa cut-off) and dialyzed extensively against HBS to remove imidazole. Antibody concentration was measured as above using the extinction coefficient of 266,525 *M*^−1^ cm^−1^. The final yield was 2.2 mg/L.

### Peptide N-glycosidase F (PNGase F) digestion

iCOMP and hCOMP were incubated either with PNGase F (1 mU/μL, Sigma-Aldrich, Cat. No.: P7367) (16 h, 37 °C) or an equal amount of 20 mM Tris (pH 7.4), 150 mM NaCl, 10 mM CaCl_2_, 0.02 % NaN_3_ and 0.05 % Brij^®^ L23 (TNC-B) buffer, before analysis by SDS-PAGE.

### COMPase assay

Purified ADAMTS variants or MMPs (12 nM) were incubated at 37 °C with 520 nM full-length COMP (corresponding to 2600 nM monomeric substrate, enzyme/monomeric substrate ratio :0.004) in TNC-B. Proteolysis was stopped by addition of Bolt^™^ LDS Sample Buffer and heating to 95 °C. Samples were frozen at −20 °C until analysis by SDS-PAGE.

### SDS PAGE and immunoblot

Samples were separated on 4–12 % SDS-PAGE Bis-Tris Plus Gels (Thermo Fisher). Proteins were transferred to nitrocellulose membranes (Bio-Rad) using a Bio-Rad Trans-Blot Turbo Transfer System at 1.3 A; up to 25 V for 30 min. Membranes were blocked for 1 h at room temperature in phosphate buffered saline (PBS), 3 % bovine serum albumin (BSA). Antibodies were diluted in PBS 3 % BSA and incubated with the blot overnight at 4 °C. The primary antibodies used were human COMP antibody (R&D Systems, AF3134, 1:5000), against the whole COMP molecule (residues Q21-A757), or neoepitope (QQS^77^) antibody (0.5 ug/mL). As secondary antibodies, rabbit anti-goat-HRP (Sigma Aldrich, 1/160,000) and goat anti-rabbit-HRP (1/10,000) (Dako Denmark A/S) were used respectively. Immunoblots were developed with Immobilon Chemiluminescent HRP substrate (Merck Millipore) with a Chemidoc Touch Imaging system (Bio-Rad). Densitometric analysis of all anti-QQS^77^ reactive bands on non-reduced blots was performed using Image lab software version 5.2.1 (Bio-Rad) using sequential exposures to avoid saturation artifacts.

Figures for unprocessed blots and gels are also included in Supplementary Figs. 10–23 with highlighted regions (in red box) corresponding to the data reported in the main manuscript figures.

#### Neoepitope antibody generation.

The neo-epitope antibody specifically detecting the COMP cleavage product ending QQS^77^ was generated by Thermo Fisher Custom antibody services. Briefly, the peptide CGGGGMQQS^77^ was fused to keyhole limpet hemocyanin for immunization of two rabbits. The same peptide was covalently coupled to a matrix for purification (positive selection) of the antibodies. The control peptide CGGGGMQQSK coupled to a matrix was used to remove antibodies that bind to uncleaved COMP (negative selection). Affinity-purified anti-TAF^577^ or anti-^578^NGV neoepitope polyclonal rabbit antibodies [56] were similarly generated by Genscript (Piscataway, NJ, USA) using as immunogens peptides CGGGGYTAF and NGVDFGGGC, respectively.

### Versicanase assay

The versicanase assay was performed as before [60,91]. ADAMTS4 (5.5 nM) and its variants were incubated with versican V1–5GAG (50 nM). At different time points (0–20 min), sub-samples were removed and reactions were stopped with EDTA. Maxisorp plates (VWR, Lutterworth, UK) were coated with 5 μg/mL anti-DPEEAE neoepitope antibody (Cat n. PA1–1748A, Life Technologies, Paisley, UK) in carbonate buffer pH 9.6 (16 h, 4 °C). This neoepitope antibody specifically recognizes the N-terminal fragment versikine, generated when versican is cleaved at the Glu^441^-Ala^442^ bond. Washing steps were performed in triplicate with 300 μL PBS containing 0.1 % Tween-20 between each step. Plates were blocked with 3 % BSA/PBS for 2 h, at RT. The samples from the digestion experiments were diluted in 3 % BSA/PBS and added to the plate (100 μL, 2 h, 25 °C). Bound versikine fragments were detected using anti-G1 monoclonal antibody (Cat. No. ab171887, Abcam, Cambridge, UK) (3 μg/mL in 0.5 % BSA/PBS, 1.5 h, 25 °C), followed by horseradish peroxidase (HRP)-conjugated anti-mouse antibodies (Cat. No. P044701–2, Agilent Technologies LTD, Cheadle, UK) (2.4 μg/mL, 1 h, RT). The assay was developed by addition of o-phenylenediamine dihydrochloride (OPD, Cat. No. 34,006, Sigma Aldrich, Gillingham, UK) for 10 min and reactions were stopped with 2 M H_2_SO_4_. The absorbance was read at 492 nm using a SpectraMax i3x Multi-Mode Detection Platform (Molecular Devices). For each dilution, the amount of neoepitope generated was derived from a standard curve (0–1.56 nM) of versican V1–5GAG completely digested with purified recombinant ADAMTS5. The catalytic efficiency, *k*_cat_/*K*_m_, was determined using GraphPad Prism software (version 8.4) by fitting the data from the time-course reactions into the equation, *P* = 1 − exp(−1 × [ADAMTS4] × *t* × *k*_cat_/*K*_m_), where P is the fraction of cleaved versican and t is the time in seconds [90].

### QF peptide cleavage assay

Antibody rec7E8.1E3 (0–8 nM) and ADAMTS4 (0.2 nM) were incubated in TNC-B for 1 h at 37 °C before addition of the QF peptide substrate fluorescein-5(6)-carbonyl-Ala-Glu~Leu-Asn-Gly-Arg-Pro-Ile-Ser-Ile-Ala-Lys(5(6)-tetramethyl-6-carboxyrhodamine (Fam-AE~LQGRPISIAK-TAMRA) (custom synthesized by Bachem) at a final concentration of 3.5 μM in 384 black wells (Greiner bio-one, Cat. No 784,900) (total volume: 20 μL) [92]. Substrate cleavage was monitored on a SpectraMax i3x Multi-Mode Detection Platform at an excitation wavelength of 485 nm and an emission wavelength of 538 nm (495 nm cut-off) for 2 h at 37 °C. Data were fitted to the Morrison equation for tight binding inhibitors [93] on GraphPad Prism 8.4:

$$v_{i}/v_{0}=1-\left(\left({\left[\text{E}\right]}_{\text{t}}+\left[\text{I}\right]+K_{\text{i}\;\text{app}}\right)-\surd \left({\left({\left[\text{E}\right]}_{\text{t}}+\left[\text{I}\right]+K_{\text{i}\;\text{app}}\right)}^{2}-4{\left[\text{E}\right]}_{\text{t}}\left[\text{I}\right]\right)/2{\left[\text{E}\right]}_{\text{t}}\right)$$

where [E]_t_ is the total active enzyme concentration.

### Label-free quantitative proteomics analysis by LC-MS/MS

Approximately 6 μg total ADAMTS-digested purified hCOMP (ADAMTS4/COMP ratio: 0.002) was lyophilized in a SpeedVac evaporator, reconstituted in 50 μL 6 M urea, 100 mM Tris, pH 7.0, reduced with 10 mM dithiothreitol and alkylated using 40 mM iodoacetamide, followed by quenching with 40 mM dithiothreitol. The urea concentration was reduced to 1.2 M by diluting the sample with 160 μL double-distilled water and the pH was adjusted to >8.0 with 100 mM ammonium bicarbonate. Samples underwent overnight digestion with trypsin at an enzyme/protein ratio of 1:7 at 37 °C. The tryptic peptide mixture was desalted using Pierce C18 spin columns (Thermo Fisher Scientific), lyophilized in a SpeedVac evaporator, reconstituted in 30 μL 1 % acetic acid, and separated on a Dionex 15 cm × 75 μm id Acclaim Pepmap C18, 2μm, 100 Å reversed phase capillary chromatography column. Peptides were eluted using buffers A (0.1 % formic acid in water) and B (0.1 % formic acid in acetonitrile) sequentially with a stepwise gradient. After sample loading over 5 min with 2 % buffer B, elution was performed with a continuous gradient of 2 %–40 % buffer B from 5 to 110 min, followed by a continuous gradient of 40 %–80 % buffer B over 5 min, and finally with 80 % buffer B for an additional 5 min. Five μL volumes of the samples were injected and the peptides eluted from the column were introduced into the source of an Orbitrap Fusion Lumos mass spectrometer (ThermoFisher) in-line. The nanospray ion source was operated at 1.9 kV and peptides were analyzed using data-dependent collision-induced dissociation. MS2 spectra were searched against the UniProtKB/Swiss-Prot human database using Proteome Discoverer 2.2. Carboxyamidomethylation of cysteine was set as a fixed modification and oxidation of methionine was the variable modification. The search included semi-tryptic peptides and allowed for up to 3 missed tryptic cleavages. Proteome Discover 2.2 was used for label-free quantitation of peptide abundance. The active enzyme/inactive enzyme peptide abundance ratio was determined for each unique peptide and *z*-scores calculated. Supporting evidence for cleavage sites was investigated by manually searching for overlapping tryptic peptides. All enumeration is based on NCBI reference sequence NP_000086.2, Cartilage Oligomeric Matrix Protein precursor [*Homo sapiens*].

The mass spectrometry proteomics data have been deposited to the ProteomeXchange Consortium via the PRIDE [94] partner repository with the dataset identifier PXD057263 and 10.6019/PXD057263.

### Determination of ADAMTS4 cleavage site specificity

A list of ADAMTS4 substrates was initially downloaded from the MEROPS database (https://www.ebi.ac.uk/merops/index.shtml) [79] under identifier M12.221 and manually analyzed to remove non- ECM substrates and redundant cleavage sites in orthologue proteins. Cleavage sites were manually annotated to reflect UniProt identifiers instead of traditional nomenclature and original references were inspected. When appropriate, cleavage sites not attributed to ADAMTS4 in the original papers were removed and missing cleavage sites were added. Finally, the cleavage sites identified in the present study were added. The final list (Supplementary Table 1), comprising 114 distinct cleavage sites in 17 substrates, was uploaded on the IceLogo web server (https://iomics.ugent.be/icelogoserver) [95] for generation of logos against a pre-compiled Swiss-Prot composition reference set from *Homo sapiens*. The following parameters were selected: scoring system: percentage (*i.e*., comparing the frequency percentage of an amino acid at a certain location in the multiple sequence alignment and the reference set); p value: 0.05.

### Molecular modeling

The prediction of an interaction between mature ADAMTS4 and the pentameric COMP NTD was performed using the ColabFold pipeline [67], using the AlphaFold2 network for multimers [66,96]. Multiple sequence alignments were obtained with MMseqs2 [97], using both paired and unpaired alignments for the complexes. Six recycling cycles were used during the prediction runs. The sequences for COMP and ADAMTS4 were obtained from UniProt [98] (UniProt ID P49747 and O75173, respectively). The input sequences provided were five chains of the COMP sequence (G^22^-R^86^) and mature ADAMTS4 (R^218^-K^837^). Outputs were visualized using PAE Viewer [99]. The assessment of the top 5 predicted models (provided as Supplementary pdb files 1–5) based on the Local Distance Difference Test (lDDT), reveal that all models achieved scores above 80, which are deemed to be optimal (Supplementary Fig. 24). However, some regions had lower scores, indicating a high degree of flexibility. Consequently, the model which scored the highest was selected for further analysis. Figures were generated using visualized using PyMOL, version 2.5.4.

### Detection of COMP fragments released from human OA cartilage explants

Human OA cartilage was obtained from the Clatterbridge hospital following knee arthroplasty procedures with informed patient consent in full compliance with national and institutional ethical requirements, the United Kingdom Human Tissue Act, and the Declaration of Helsinki (REC 18/WA/0344). Explants (~36 mm^3^, ~40 mg wet volume/weight) from each of 4 patients were placed in one well of a 24-well plate and allowed to rest for 24 h in 1 mL of DMEM containing 10 % FBS before use. The medium was replaced, and the cartilage was rested for a further 24 h in 1 mL DMEM at 37 °C before assays. The explants were then incubated in 0.6 mL of fresh DMEM containing control IgG1 (100 nM) or rec7E8.1E3 anti-ADAMTS4 antibody (10 or 100 nM). After 24 h incubation, the medium was collected, and proteins were precipitated with trichloroacetic acid. The resultant precipitates were dissolved in SDS sampling buffer and subjected to SDS-PAGE followed by immunoblotting analysis.

### Immunohistochemistry

Paraffin embedded sections (5 μm thickness) of human OA cartilage explants were fixed in neutral buffered formalin and used for immunohistochemical detection of ADAMTS4 and the COMP QQS^77^ neoepitope. Initially, slides were rehydrated with serial incubations in Histoclear II (Merck), 100 %, 90 % and 70 % ethanol prior to heat-induced antigen retrieval at 95 °C (R&D systems) for 10 min. Slides were then soaked in PBS before blocking endogenous peroxidase with 3 % H_2_O_2_ for 15 min followed by a gentle wash with 0.05 % PBS-T. Avidin-biotin activity was blocked (ABC blocking kit, Vector Labs) for 30 min prior to blocking non-specific binding sites with Protein Block (Abcam, Cat. No: ab64226) for 3 h. Slides were then reacted with ADAMTS4 (ThermoFisher Scientific Cat. No.: PA1–1749A 1:500; 3 μg/mL) and QQS^77^ (1:1000; 0.48 μg/mL) primary antibodies overnight at 4 °C in a humidified chamber. Slides were thoroughly washed with 0.05 % PBS-T and then incubated with an anti-rabbit HRP conjugated secondary antibody (Agilent Technologies Cat. No P044801–2, 1:1000; 0.3 μg/mL) for 1 h. To enhance detection, tissues were overlayed with ABC—HRP solution (Vector Labs) for 10 min, washed, and then visualized with DAB (ThermoFisher Cat. No 34,002). Lastly, slides were dehydrated in the reverse order of hydration before mounting in Histomount (Scientific Laboratory supplies Cat. No NAT1308). Negative controls were incubated with secondary antibodies only (data not shown).

## Statistical analysis

Statistical analysis was performed on GraphPad Prism software (version 8.4). When comparing 2 groups, unpairing Student t-test (two-tailed) with Mann-Whitney correction was applied. When comparing >2 groups, one-way ANOVA with Tukey correction for multiple comparisons was applied. *p* < 0.05 was considered statistically significant.

## Supplementary Material

De Groot 2025 Supplement

## Figures and Tables

**Table 1:** Significant peptides from ADAMTS4 digestion of hCOMP.

Semi-tryptic peptide sequence	Corresponding spanning tryptic peptide sequence	Semi-tryptic peptide				Corresponding spanning tryptic peptide		
		Cleavage site position	Region	Protease/control	z score	Position	Control/protease	z score
**[Q].S**VRTGLPSVRPLLHCAPGFCFPGVACIQTESGAR.[C]	[R].EITFLKNTVMECDACGMQ**QSV**R.[T]	Q76-S77	NTD	100	3.37	58–79	71.3	2.4
**[S].V**RTGLPSVRPLLHCAPGFCFPGVACIQTESGAR.[C]	[R].EITFLKNTVMECDACGMQQ**SV**R.[T]	S77-V78	NTD	142.61	3.63	58–79	71.3	2.4
[K].NTVMECDACGMQQ**S.[V]**	–	S77-V78	NTD	100	3.37			
[R].EITFLKNTVMECDACGMQQ**S.[V]**	–	S77-V78	NTD	100	3.37			
**[S].V**RPLLHCAPGFCFPGVACIQTESGAR.[C]		S84-V85	NTD	100	3.37			
**[A].C**IQTESGAR.[C]	–	A101-C102	EGF1	100	3.37			
[R].TGLPSVRPLLHCAPGFCFPGVACIQT**E.[S]**	–	E106-S107	EGF1	100	3.37			
[K].QVCTDINECETGQHNCVP**N.[S]**	[K].ANKQVCTDINECETGQHNCVP**NS**VCINTR.[G]	N194-S195	EGF3	100	3.37	173–201	738	3.92
**[C].P**DGSPSECHEHADCVLER.[D]	[R].AQRF**CP**DGSPSECHEHADCVLERDGSR.[S]	C229-P230	EGF3	100	3.37	223–249	100	2.65
[R].SCVCAVGWAGNGILCGR**D.[T]**	–	D269-T270	TSP1	100	3.37			
**[D].N**CVTVPNSGQEDVDRDGIGDACDPDADGDGVPNEKDNCPLVR.[N]	[K].**DN**CVTVPNSGQEDVDR.[D]	D290-N291	TSP1	100	3.37	290–305	100	3.22
**[C].V**TVPNSGQEDVDRDGIGDACDPDADGDGVPNEKDNCPLVR.[N]	[K].DN**CV**TVPNSGQEDVDR.[D]	C292-V293	TSP1	100	3.37	290–305	100	3.22
**[D].V**DRDGIGDACDPDADGDGVPNEKDNCPLVR.[N]	–	D302-V303	TSP2	100	3.37			
[R].KDNCVTVPNSGQEDVDRDGIG**D.[A]**	[R].DGIG**DA**CDPDADGDGVPNEKDNCPLVR.[N]	D310-A311	TSP2	100	3.37	306–332	100	2.65
	[R].DGIG**DA**CDPDADGDGVPNEKDNCPLVRNPDQR.[N]	D310-A311	TSP2			306–337	100	2.65
**[A].C**DPDADGDGVPNEKDNCPLVR.[N]	[R].DGIGD**AC**DPDADGDGVPNEKDNCPLVR.[N]	A311-C312	TSP2	100	3.37	306–332	100	2.65
	[R].DGIGD**AC**DPDADGDGVPNEKDNCPLVRNPDQR.[N]	A311-C312	TSP2			306–337	100	2.65
**[P].D**ADGDGVPNEKDNCPLVR.[N]	[R].DGIGDACD**PD**ADGDGVPNEKDNCPLVR.[N]	P314-D315	TSP2	100	3.37	306–332	100	2.65
	[R].DGIGDACD**PD**ADGDGVPNEKDNCPLVRNPDQR.[N]	P314-D315	TSP2			306–337	100	2.65
**[A].D**GDGVPNEKDNCPLVR.[N]	[R].DGIGDACDPD**AD**GDGVPNEKDNCPLVR.[N]	A316-D317	TSP2	100	3.37	306–332	100	2.65
**[A].D**GDGVPNEKDNCPLVR.[N]	[R].DGIGDACDPD**AD**GDGVPNEKDNCPLVRNPDQR.[N]	A316-D317	TSP2			306–337	100	2.65
**[G].V**PNEKDNCPLVR.[N]	[R].DGIGDACDPDADGD**GV**PNEKDNCPLVR.[N]	G320-V321	TSP2	100	3.37	306–332	100	2.65
	[R].DGIGDACDPDADGD**GV**PNEKDNCPLVRNPDQR.[N]	G320-V321	TSP2			306–337	100	2.65
[K].DNCVTVPNSGQEDVDRDGIGDACDPDADGDGVP**N.[E]**	[[R].DGIGDACDPDADGDGVP**NE**KDNCPLVR.[N]	N323-E324	TSP2	100	3.37	306–332	100	2.65
	[R].DGIGDACDPDADGDGVP**NE**KDNCPLVRNPDQR.[N]	N323-E324	TSP2			306–337	100	2.65
**[N].P**DQADVDHDFVGDACDSDQDQDGDGHQDSR.[D]	–	N415-P416	TSP5	100	3.37			
**[D].G**VGDVCQDDFDADKVVDKIDVCPENAEVTLTDFR.[A]	[R].**DG**VGDVCQDDFDADKVVDK.[I]	D498-G499	TSP8	100	3.37	498–512	100	2.64
[R].LVPNPGQEDADRDGVG**D.[V]**	[R].DGVG**DV**CQDDFDADKVVDK.[I]	D502-V503	TSP8	100	3.37	498–512	100	2.64
[K].IDVCPENAEVTL**T.[D]**	–	T529-D530	CTD	100	3.37			
**[F].R**AFQTVVLDPEGDAQIDPNWVVLNQGR.[E]	–	F531-R532	CTD	100	3.37			
**[Q].T**VVLDPEGDAQIDPNWVVLNQGR.[E]	–	Q535-T536	CTD	100	3.37			
**[T].V**VLDPEGDAQIDPNWVVLNQGR.[E]	–	T536-V537	CTD	100	3.37			
**[N].W**VVLNQGR.[E]	–	N550-W551	CTD	100	3.37			
[R].AFQTVVLDPEGDAQIDPNWVVLN**Q.[G]**	–	Q556-G557	CTD	100	3.37			
[R].EIVQTMNSDPGLAVGYTA**F.[N]**	–	F577-N578	CTD	100	3.37			
**[E].G**PELVADSNVVLDTTMRGGR.[L]	–	E701-G702	CTD	100	3.37			
[R].FYEGPELVADSNVVLDT**T.[M]**	–	T716-M717	CTD	100	3.37			
[R].FYEGPELVADSNVVLDTT**M.[R]**	–	M717-R718	CTD	100	3.37			
[R].VRFYEGPELVADSNVVLDTT**M.[R]**	–	M717-R718	CTD	100	3.37			
[R].LGVFCF**S.[Q]**	–	S728-Q729	CTD	100	3.37			
**[Q].L**RQAFEGKPIPNPLLGLDSTR.[T]	–	Q753-L754	CTD	100	3.37			

Residues within brackets preceded or followed the detected peptide sequence (within full stops). P1 and P1′ residues are in bold. CTD, C-terminal domain; EGF, epidermal-growth factor-like repeat; NTD, N-terminal domain; TSP, thrombospondin type III repeat. *z*-score is the number of standard deviations from the mean protease/control ratio of all peptides found in both groups.

**Table 2:** Significant peptides from ADAMTS1 digestion of hCOMP.

Semi-tryptic peptide sequence	Corresponding spanning tryptic peptide sequence	Semi-tryptic peptide				Corresponding tryptic peptide		
		Cleavage site	Region	Protease/control	z score	Position	Control/protease	z score
**[Q].S**PLGSDLGPQMLRELQETNAALQDVRELLR.[Q]	–	Q23-S24	NTD	100.00	3.33			
**[L].G**SDLGPQMLRELQETNAALQDVRELLR.[Q]	–	L26-G27	NTD	100.00	3.33			
**[D].L**GPQMLRELQETNAALQDVRELLR.[Q]	–	D29-L30	NTD	100.00	3.33			
**[M].L**RELQETNAALQDVRELLR.[Q]	–	M34-L35	NTD	100.00	3.33			
[K].NTVMECDACGMQ**Q.[S]**	[R].EITFLKNTVMECDACGMQ**QS**VR.[T]	Q76-S77	NTD	100.00	3.33	58–79	100	3.1
[R].QQVREITFLKNTVMECDACGMQ**Q.[S]**	[R].QQVREITFLKNTVMECDACGMQ**QS**VR.[T]	Q76-S77	NTD	100.00	3.33	54–79	100	3.1
**[S].V**RTGLPSVRPLLHCAPGFCFPGVACIQTESGAR.[C]	[R].EITFLKNTVMECDACGMQQ**SV**R.[T]	S77-V78	NTD	230.35	3.86	58–79	100	3.1
	[R].QQVREITFLKNTVMECDACGMQQ**SV**R.[T]	S77-V78	NTD			54–79	100	3.1
[R].EITFLKNTVMECDACGMQQ**S.[V]**	–	S77-V78	NTD	102.01	3.34			
[R].QQVREITFLKNTVMECDACGMQQ**S.[V]**	–	S77-V78	NTD	100.00	3.33			
**[G].**LPSVRPLLHCAPGFCFPGVACIQTESGAR.[C]	–	G81-L82	NTD	100.00	3.33			
[R].TGLPSVRPLLHCAPGFCFPGVACIQ**T.[E**]	–	T105-E106	EGF1	100.00	3.33			
**[D].I**NECETGQHNCVPNSVCINTR.[G]	–	D180-I181	EGF3	100.00	3.33			
[K].ANKQVCTDINECETGQHNC**V.[P]**	–	V192-P193	EGF3	100.00	3.33			
[R].FCPDGSPSECHEHADCVLER**D.[G]**	[R].FCPDGSPSECHEHADCVLER**DG**SR.[S]	D248-G249	EGF4	100.00	3.33	228–251	100	3.1
–	[R].**DG**SRSCVCAVGWAGNGILCGR.[D]	D248-G249	EGF4			248–268	100	3.1
**[C].Q**DDFDADKVVDKIDVCPENAEVTLTDFR.[A]	–	C504-Q505	TSP8	100.00	3.33			
**[D].F**RAFQTVVLDPEGDAQIDPNWVVLNQGR.[E]	–	D530-F531	CTD	100.00	3.33			
[R].AFQTVVLDPEGDAQIDPNWVVLNQGREIVQTMNSDPGLAVGYT**A.[F]**	–	A576-F577	CTD	100.00	3.33			
[K].QMEQTYWQANPFR**A.[V]**	–	A629-V630	CTD	100.00	3.33			
[K].QMEQTYWQANPFRAVAEPGIQLK**A.[V]**	[K].**AV**KSSTGPGEQLRNALWHTGDTESQVR.[L]	A639-V640	CTD	100.00	3.33	639–665	150.82	3.45
**[Q].L**RQAFEGKPIPNPLLGLDSTR.[T]	-	Q753-L754	CTD	100.00	3.33			

Residues within brackets preceded or followed the detected peptide sequence (within full stops). P1 and P1′ residues are in bold. CTD, C-terminal domain; EGF, epidermal-growth factor-like repeat; NTD, N-terminal domain; TSP, thrombospondin type III repeat. *z*-score is the number of standard deviations from the mean protease/control ratio of all peptides found in both groups.

**Table 3:** Correspondence of COMP cleavage sites and peptides identified in the present study with peptides identified in cartilage or synovial fluid of patients with joint disease.

Cleavage site	Region	Semi-tryptic peptide	Protease	Peptide identified in human OA samples	Disease	Location	References
L26-G27	NTD	**[L].G**SDLGPQMLRELQETNAALQDVRELLR.[Q]	ADAMTS1	GSDLGPQMLRELQETNAALQDVRELLR	OA	C	[55,57]
D29-L30	NTD	**[D].L**GPQMLRELQETNAALQDVRELLR.[Q]	ADAMTS1	LGPQMLRELQETNAALQDVRELLR	OA	C	[55,57]
M34-L35	NTD	**[M].L**RELQETNAALQDVRELLR.[Q]	ADAMTS1	LRELQETNAALQDVRELLR	OA	C	[57]
Q76-S77	NTD	[K].NTVMECDACGMQ**Q.[S]**	ADAMTS1	NTVMECDACGMQQ	OA	C, SF	[57]
Q76-S77	NTD	**[Q].S**VRTGLPSVRPLLHCAPGFCFPGV	ADAMTS4	SVRTGLPSVRPLLHCAPGFCFPGVACIQTESGAR	OA	C	[57]
S77-V78	NTD	**[S].V**RTGLPSVRPLLHCAPGFCFPGVACIQTESGAR.[C]	ADAMTS1	NTVMECDACGMQQS	AT	C, SF	[56,57]
		**[S].V**RTGLPSVRPLLHCAPGFCFPGVACIQTESGAR.[C]		VRTGLPSVRPLLHCAPGFCFPGVACIQTE	OA	C	[57]
		[R].EITFLKNTVMECDACGMQQ**S.[V]**		EITFLKNTVMECDACGMQQS	OA	SF	[57]
		[R].QQVREITFLKNTVMECDACGMQQS**.[V]**					
		**[S].V**RTGLPSVRPLLHCAPGFCFPGVACIQTESGAR.[C]					
S77-V78	NTD	**[S].V**RTGLPSVRPLLHCAPGFCFPGVACIQTESGAR.[C]	ADAMTS4	NTVMECDACGMQQS	AT	C, SF	[55]
		**[S].V**RTGLPSVRPLLHCAPGFCFPGVACIQTESGAR.[C]	ADAMTS4	VRTGLPSVRPLLHCAPGFCFPGVACIQTESGAR	OA	C, SF	[55]
		[K].NTVMECDACGMQQ**S.[V]**		VRTGLPSVRPLLHCAPGFCFPGVACIQTESGAR	OA*	C	[55]
		[R].EITFLKNTVMECDACGMQQ**S.[V]**					
G81-L82	NTD	**[G].L**PSVRPLLHCAPGFCFPGVACIQTESGAR.[C]	ADAMTS1	LPSVRPLLHCAPGFCFPGVACIQTESGAR	OA	C	[55,57]
S84-V85	NTD	**[S].V**RPLLHCAPGFCFPGVACIQTESGAR.[C]	ADAMTS4	VRPLLHCAPGFCFPGVACIQTESGAR	OA	C	[57]
A101-C102	EGF1	**[A].C**IQTESGAR.[C]	ADAMTS4	CIQTESGAR	OA	C, SF	[57]
D180-I181	EGF3	**[D].I**NECETGQHNCVPNSVCINTR.[G]	ADAMTS1	INECETGQHNCVPNSVCINTR	OA	C	[55,57]
V192-P193	EGF3	[K].ANKQVCTDINECETGQHNC**V.[P]**	ADAMTS1	ANKQVCTDINECETGQHNCV	OA	C	[57]
N194-S195	EGF3	[K].QVCTDINECETGQHNCVP**N.[S]**	ADAMTS4	SVCINTRGSF	RA	SF	[55,56]
		[K].QVCTDINECETGQHNCVP**N.[S]**	ADAMTS4	QVCTDINECETGQHNCVPN	OA	SF	[57]
		[K].QVCTDINECETGQHNCVP**N.[S]**	ADAMTS4	SVCINTR	OA	C	[56]
C229-P230	EGF3	**[C].P**DGSPSECHEHADCVLER.[D]	ADAMTS4	PDGSPSECHEHADCVLER	OA	C, SF	[55,57]
D269-T270	TSP1	[R].SCVCAVGWAGNGILCGR**D.[T]**	ADAMTS4	SCVCAVGWAGNGILCGRD	OA	C, SF	[55,57]
D290-N291	TSP1	**[D].N**CVTVPNSGQEDVDRDGIGDACDPDADG	ADAMTS4	NCVTVPNSGQEDVDRDGIGDACDPDADG	OA	C, SF	[57]
D310-A311	TSP2	[R].KDNCVTVPNSGQEDVDRDGIG**D.[A]**	ADAMTS4	KDNCVTVPNSGQEDVDRDGIGD	OA	C, SF	[55,57]
A311-C312	TSP2	**[A].C**DPDADGDGVPNEKDNCPLVR.[N]	ADAMTS4	CDPDADGDGVPNEKDNCPLVR	OA	C	[55,57]
A316- D317	TSP2	**[A].D**GDGVPNEKDNCPLVR.[N]	ADAMTS4	DGDGVPNEKDNCPLVR	OA	SF	[55]
G320-V321	TSP2	**[G].V**PNEKDNCPLVR.[N]	ADAMTS4	VPNEKDNCPLVRNPDQR	OA	C	[57]
D498-G499	TSP8	**[D].G**VGDVCQDDFDADKVVDKIDVCPENAEVTLTDFR.[A]	ADAMTS4	GVGDVCQDDFDADKVVDKIDVCPENAEVTLTDFR	OA	C	[57]
D502-V503	TSP8	[R].LVPNPGQEDADRDGVG**D.[V]**	ADAMTS4	LVPNPGQEDADRDGVGD	OA	C	[57]
C504-Q505	TSP8	**[C].Q**DDFDADKVVDKIDVCPENAEVTLTDFR.[A]	ADAMTS1	QDDFDADKVVDKIDVCPENAEVTLTDFR	OA	C	[57]
F531-R532	CTD	**[F].R**AFQTVVLDPEGDAQIDPNWVVLNQGR.[E]	ADAMTS4	IDVCPENAEVTLTDF	OA, AT	SF	[56,57]
F577-N578	CTD	[R].EIVQTMNSDPGLAVGYTA**F.[N]**	ADAMTS4	EIVQTMNSDPGLAVGYTAF	OA	SF	[56]
A629-V630	CTD	[K].QMEQTYWQANPFR**A.[V]**	ADAMTS1	QMEQTYWQANPFRA	OA	C, SF	[55,57]
A639-V640	CTD	[K].QMEQTYWQANPFRAVAEPGIQLK**A.[V]**	ADAMTS1	QMEQTYWQANPFRAVAEPGIQLKA	OA	C	[55,57]
E701-G702	CTD	**[E].G**PELVADSNVVLDTTMRGGR.[L]	ADAMTS4	GPELVADSNVVLDTTMRGGR	OA	C	[57]

Residues within brackets preceded or followed the detected peptide sequence (within full stops). P1 and P1′ residues are in bold. AT, acute trauma; C, cartilage; CTD, C-terminal domain; NTD, N-terminal domain; OA, osteoarthritis; RA, rheumatoid arthritis; synovial fluid, SF.

*, indicates cleavage by HtrA1.

## Data Availability

Data will be made available on request.

## Abbreviations:

ADAMTS: a disintegrin and metalloproteinase with thrombospondin motifs
CBB: coomassie brilliant blue
COMP: Cartilage Oligomeric Matrix Protein
CR: cysteine-rich
CTD: C-terminal domain
Dis: disintegrin-like domain
ECM: extracellular matrix
EGF: epidermal-growth factor
iCOMP: *Drosophila melanogaster* S2-expressed human COMP
IL: interleukin
hCOMP: mammalian cell-expressed human COMP
MMP: matrix metalloproteinase
Mp: metalloproteinase domain
NTD: N-terminal domain
OA: osteoarthritis
QF: quenched-fluorescent peptide
RA: rheumatoid arthritis
Sp: spacer
TSP: thrombospondin type III repeat
TSR: thrombospondin type 1 repeat
WT: wild-type
