# Supplementary material for: Cleavage of Cartilage Oligomeric Matrix Protein (COMP) by ADAMTS4 generates a neoepitope associated with osteoarthritis and other forms of degenerative joint disease
Source: Matrix Biol. Author manuscript; Available in PMC 2025 Aug 12. (PMC12342048; doi:10.1016/j.matbio.2024.12.005)

**Cleavage of Cartilage Oligomeric Matrix Protein (COMP) by ADAMTS4 generates a neoepitope associated with osteoarthritis and other forms of degenerative joint disease**

Rens de Groot,^1^* Patricia Badía Folgado,^2^ Kazuhiro Yamamoto,^3^ Daniel R. Martin,^4^ Christopher D. Koch,^4^ Danielle Debruin,^5^ Sophie Blagg,^2^ Alexander F. Minns,^5^ Sumit Bhutada,^4^ Josefin Ahnström,^2^ Jonathan Larkin,^6,7^ Anders Aspberg,^8^ Patrik Önnerfjord,^8^ Suneel S. Apte,^4^ and Salvatore Santamaria^2,5^*

^1^ Institute of Cardiovascular Science, University College London, 51 Chenies Mews, London WC1E 6HX, United Kingdom;

^2^Department of Immunology and Inflammation, Imperial College London, Du Cane Road, W12 0NN, London, United Kingdom;

^3^Institute of Life Course and Medical Sciences, Faculty of Health and Life Sciences, University of Liverpool, 6 West Derby Street, Liverpool L7 8TX, United Kingdom;

^4^Department of Biomedical Engineering, Cleveland Clinic Lerner Research Institute, Cleveland, OH 44195, USA;

^5^Department of Biochemical Sciences, School of Biosciences, Faculty of Health and Medical Sciences, Edward Jenner Building, University of Surrey, Guildford, Surrey GU2 7XH, United Kingdom;

^6^SynOA Therapeutics, Philadelphia, PA, USA;

^7^Research Unit of Health Sciences and Technology, Faculty of Medicine, University of Oulu, Oulu, Finland;

^8^Rheumatology and Molecular Skeletal Biology, Department of Clinical Sciences Lund, Lund University, Lund, Sweden

* to whom the correspondence should be addressed:

Rens de Groot, email: Institute of Cardiovascular Science, University College London, 51 Chenies Mews, London WC1E 6HX, United Kingdom; [r.degroot@ucl.ac.uk](mailto:r.degroot@ucl.ac.uk); Salvatore Santamaria, Department of Biochemical Sciences, School of Biosciences, Faculty of Health and Medical Sciences, Edward Jenner Building, University of Surrey, Guildford, Surrey GU2 7XH, United Kingdom; [s.santamaria@surrey.ac.uk](mailto:s.santamaria@surrey.ac.uk)

**Supplementary Figure 1. The presence of ZnCl_2_ does not affect the ability of ADAMTS7 to cleave COMP.** hCOMP (520 nM) was incubated with ADAMTS4, ADAMTS7 (each at 10 nM) or buffer alone (0) either in the presence or absence of 2 mM ZnCl_2_ for 2 h at 37 ℃. Samples were then subjected to SDS-PAGE under non-reducing conditions and probed either with a polyclonal anti-COMP antibody **(A)** or a neoepitope anti-QQS^77^ antibody **(B)**. CF, cleavage fragment; CM, cleaved monomer; D, dimer; IB, immunoblot; M, monomer; P, pentamer. Blots representative of two independent experiments.


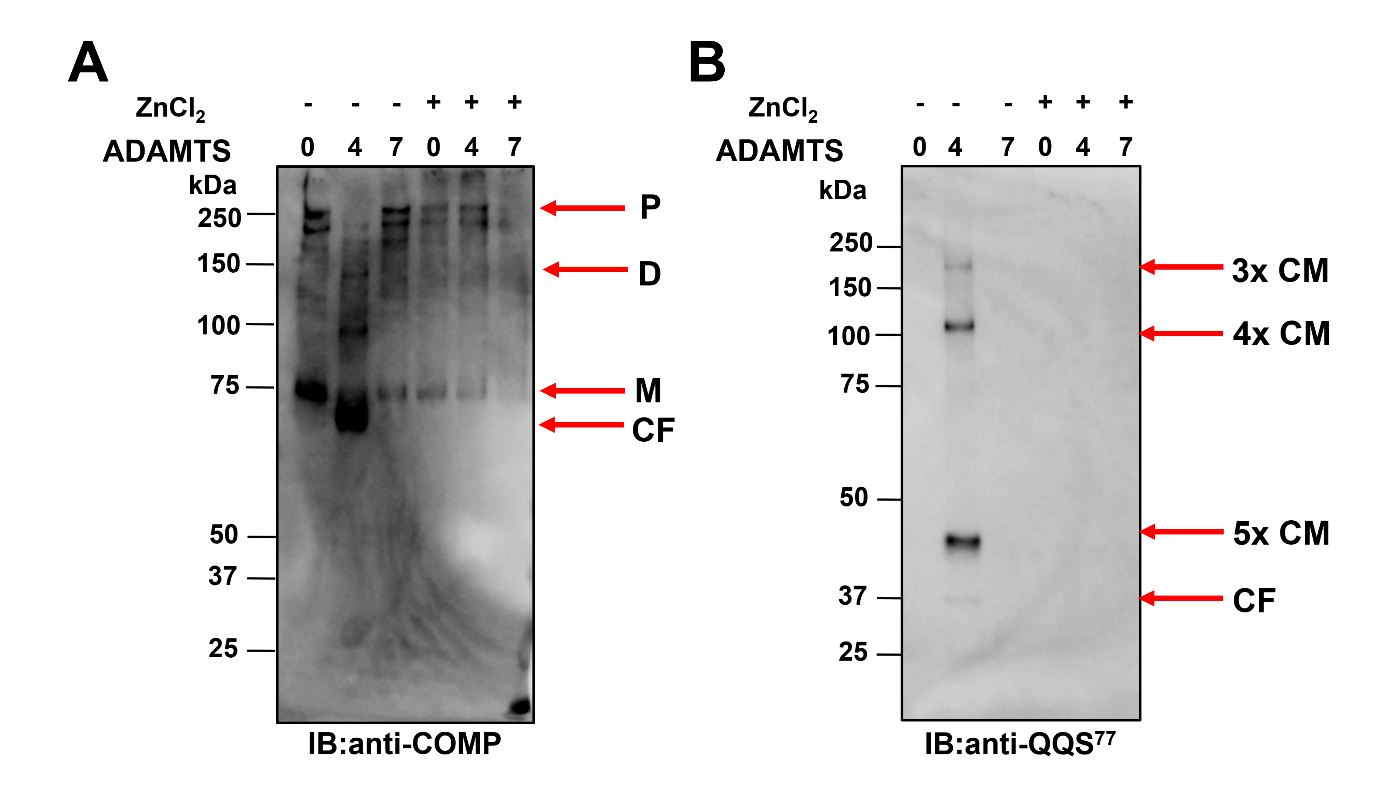


**Supplementary Figure 2. Annotated MS/MS spectra of representative ADAMTS4- derived peptides from the hCOMP digestion.** Parent ions were selected for collision-induced dissociation fragmentation at 35% collisional energy. This fragmentation creates breakages at the amino-carboxyl bond to generate a series of b-ions (which retain the original N-terminus, in blue) and y-ions (which retain the original C-terminus, in red). The m/z of the b/y ions corresponds to known amino acid residues or amino acid ensembles, providing the parent ion amino acid sequence (labelled on each spectrum).

**
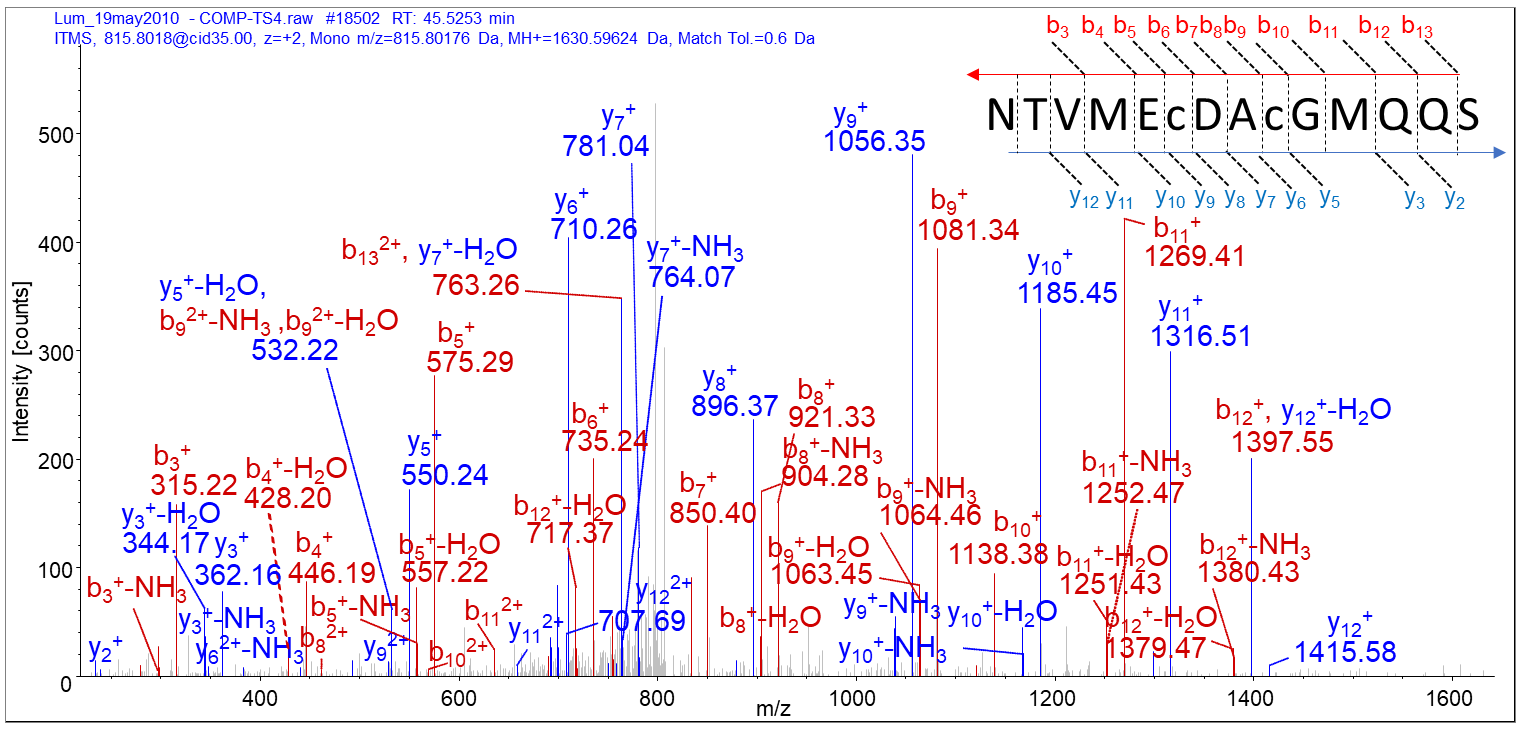
**

**
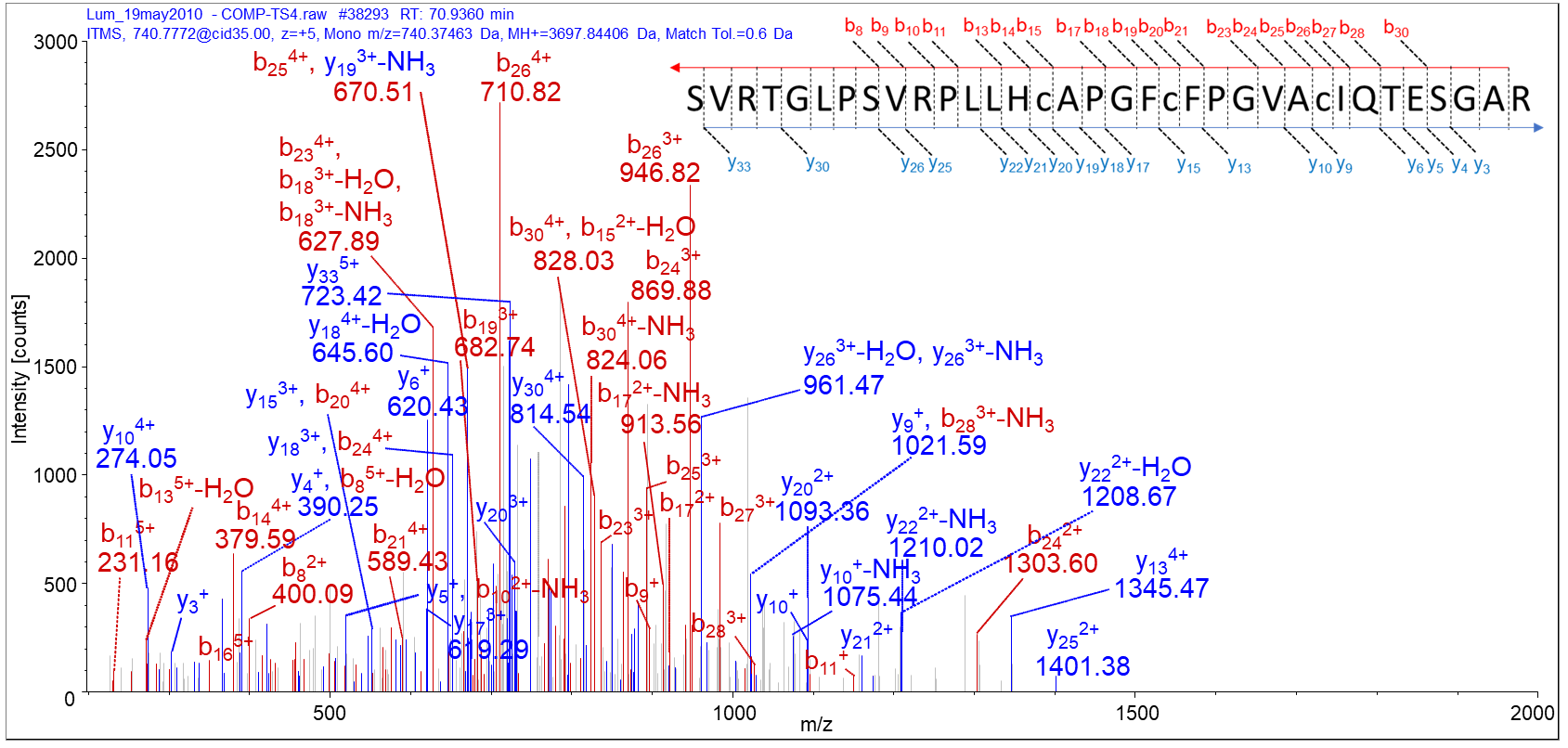
**

**
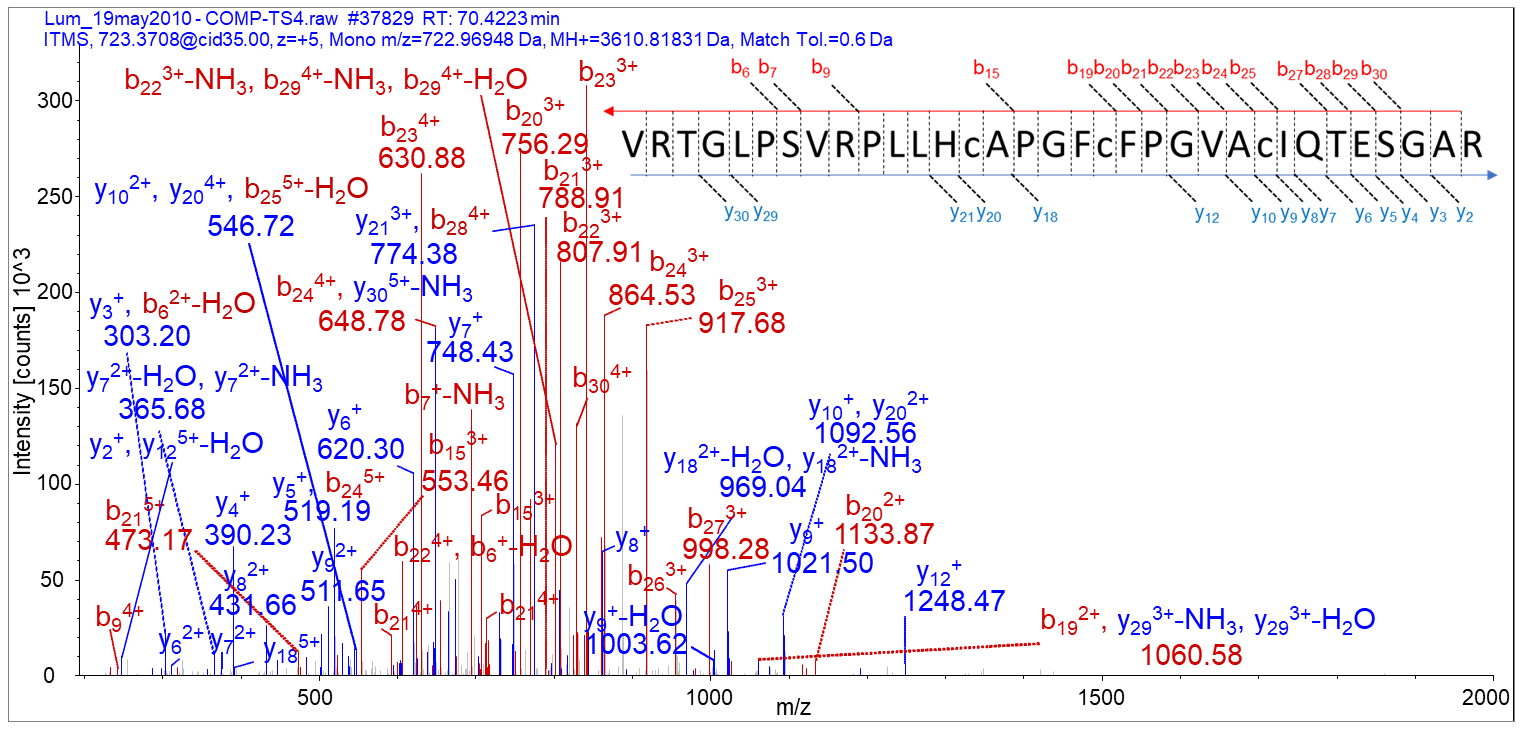
**

**
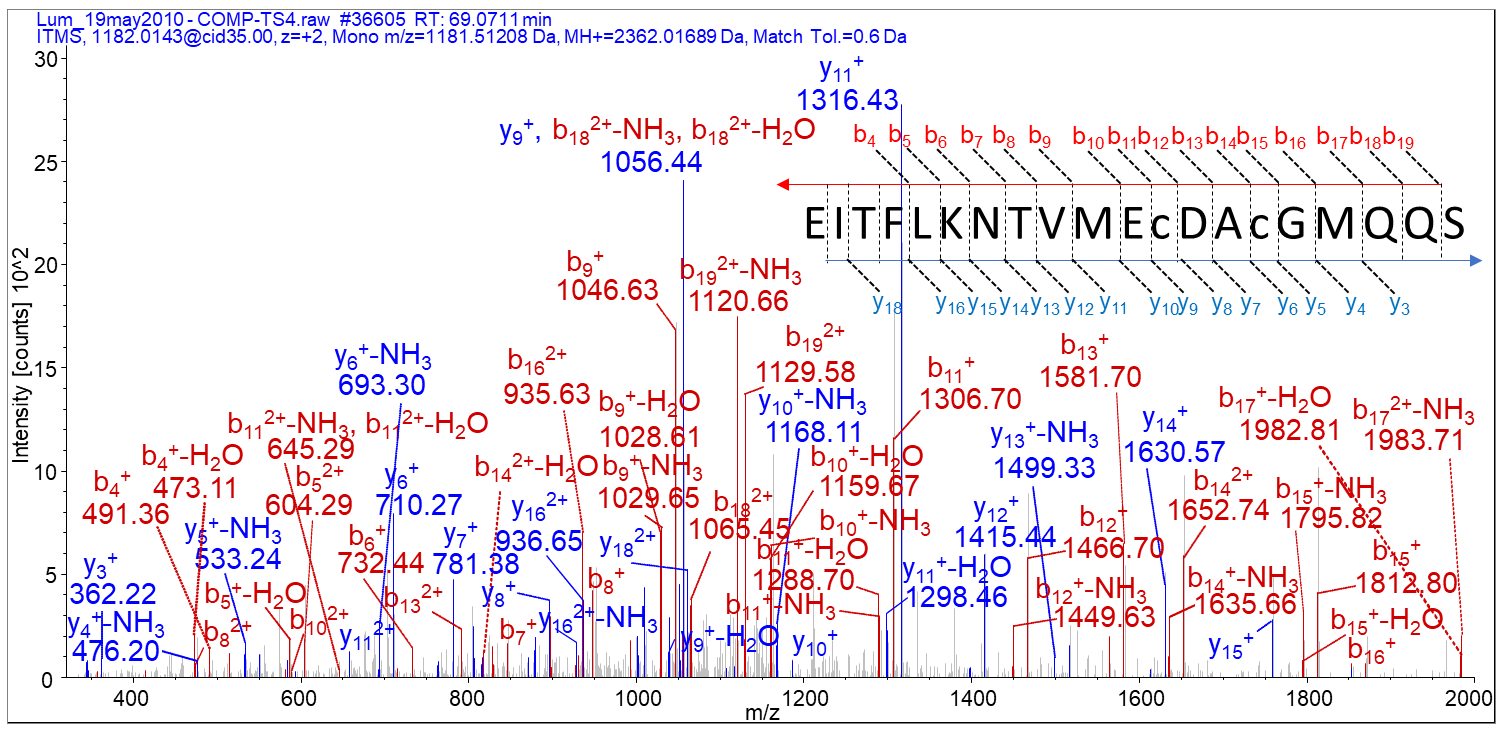
**

**
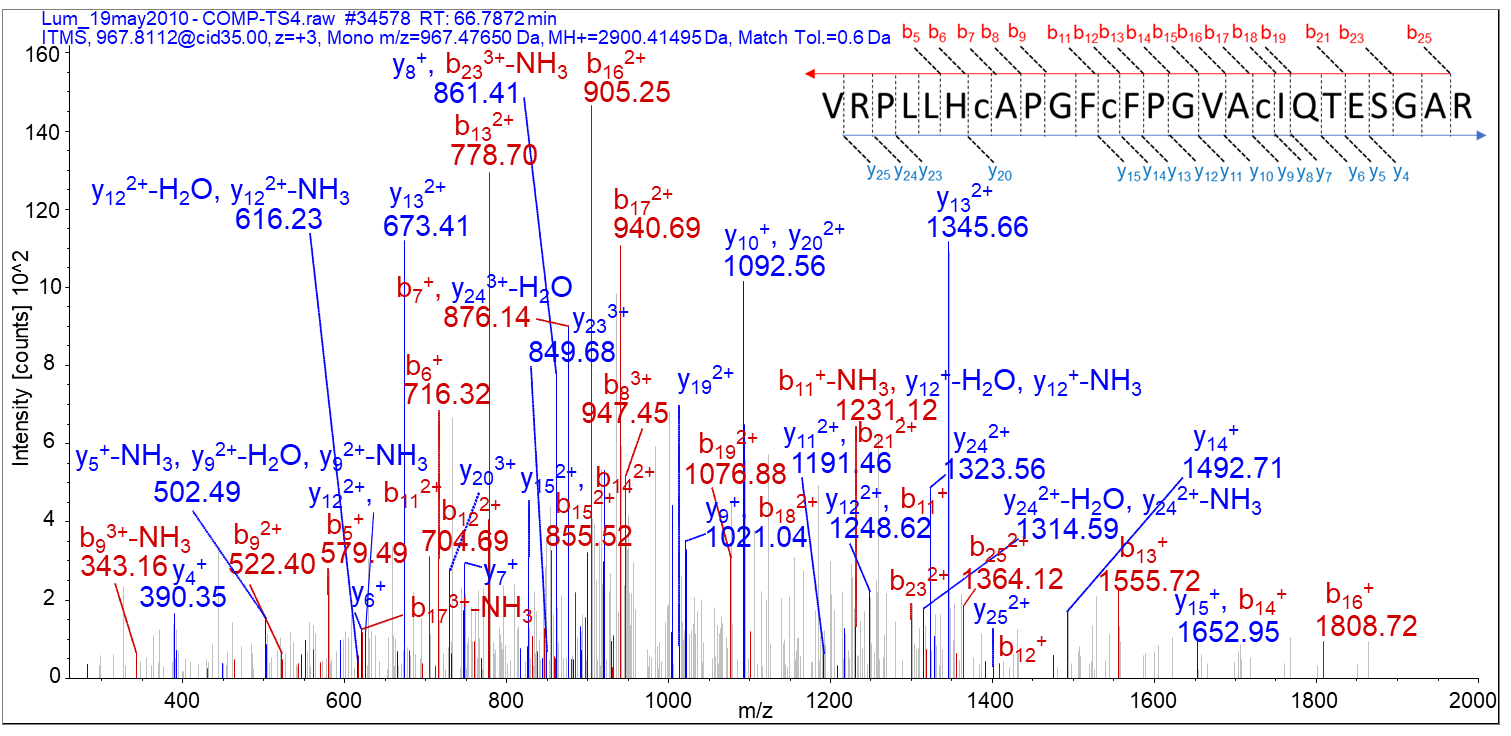
**

**Supplementary Figure 3: significant tryptic peptides more abundant in ADAMTS4 (A) and ADAMTS1 (B) EQ digests than in WT digests.**

**
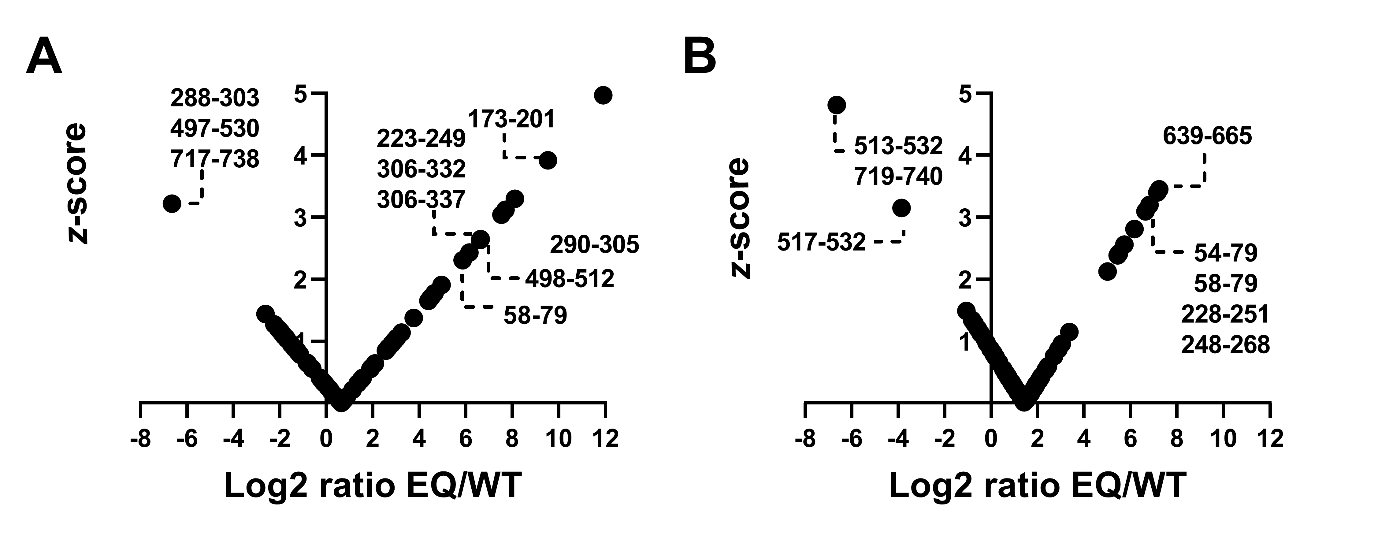
**

**Supplementary Figure 4. Annotated MS/MS spectra of representative ADAMTS1- derived peptides from the hCOMP digestion.** Parent ions were selected for collision-induced dissociation fragmentation at 35% collisional energy. This fragmentation creates breakages at the amino-carboxyl bond to generate a series of b-ions (which retain the original N-terminus, in blue) and y-ions (which retain the original C-terminus, in red). The m/z of the b/y ions corresponds to known amino acid residues or amino acid ensembles, providing the parent ion amino acid sequence (labelled on each spectrum).

**
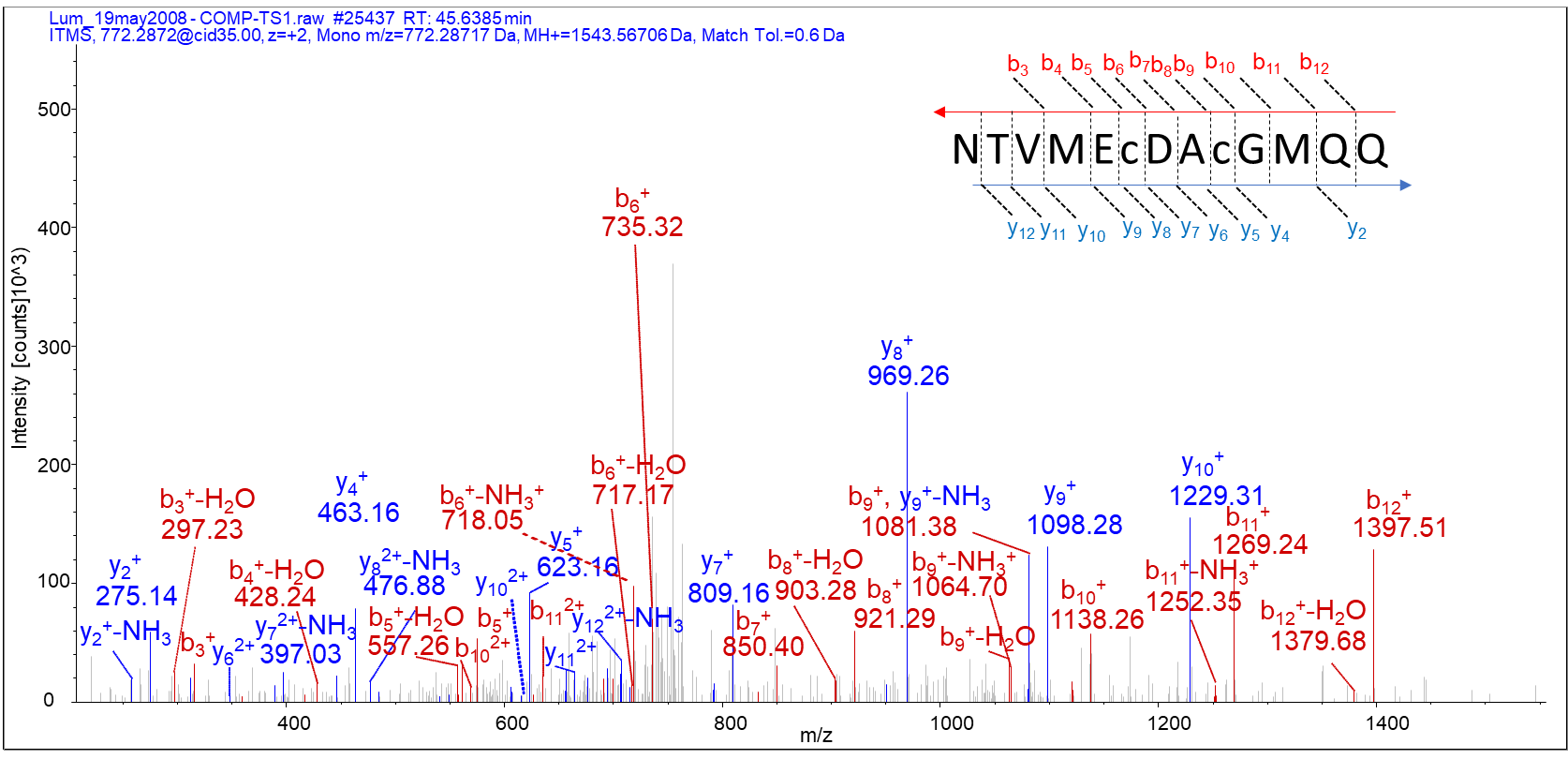
**

**
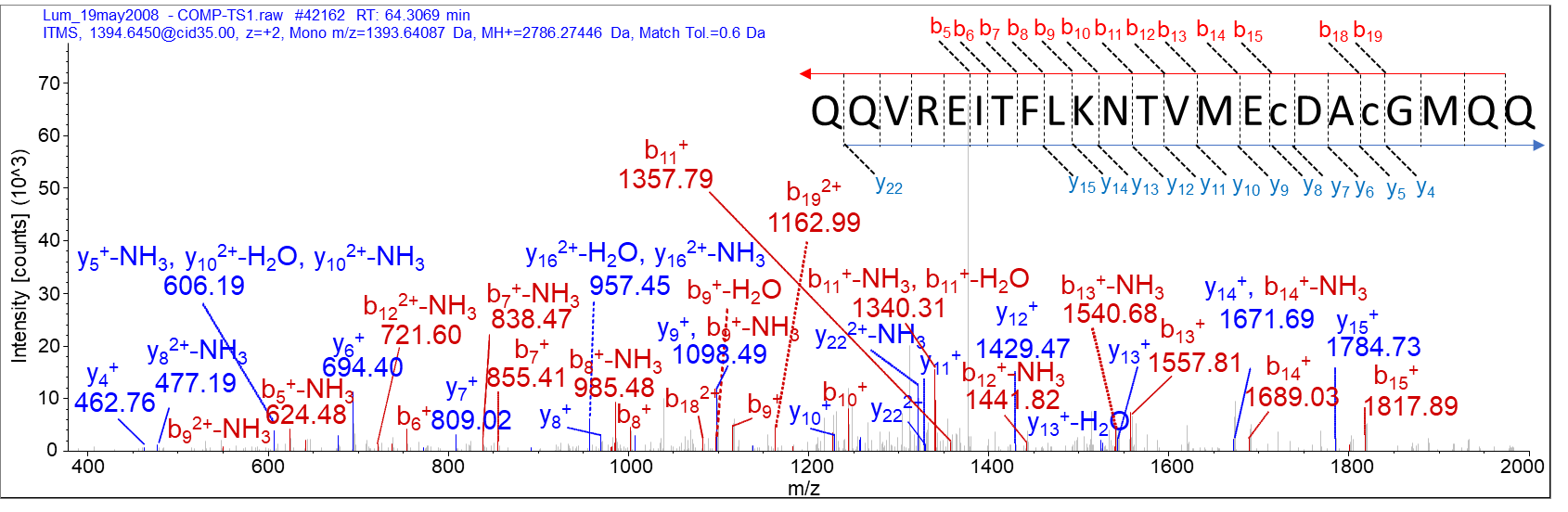
**

**
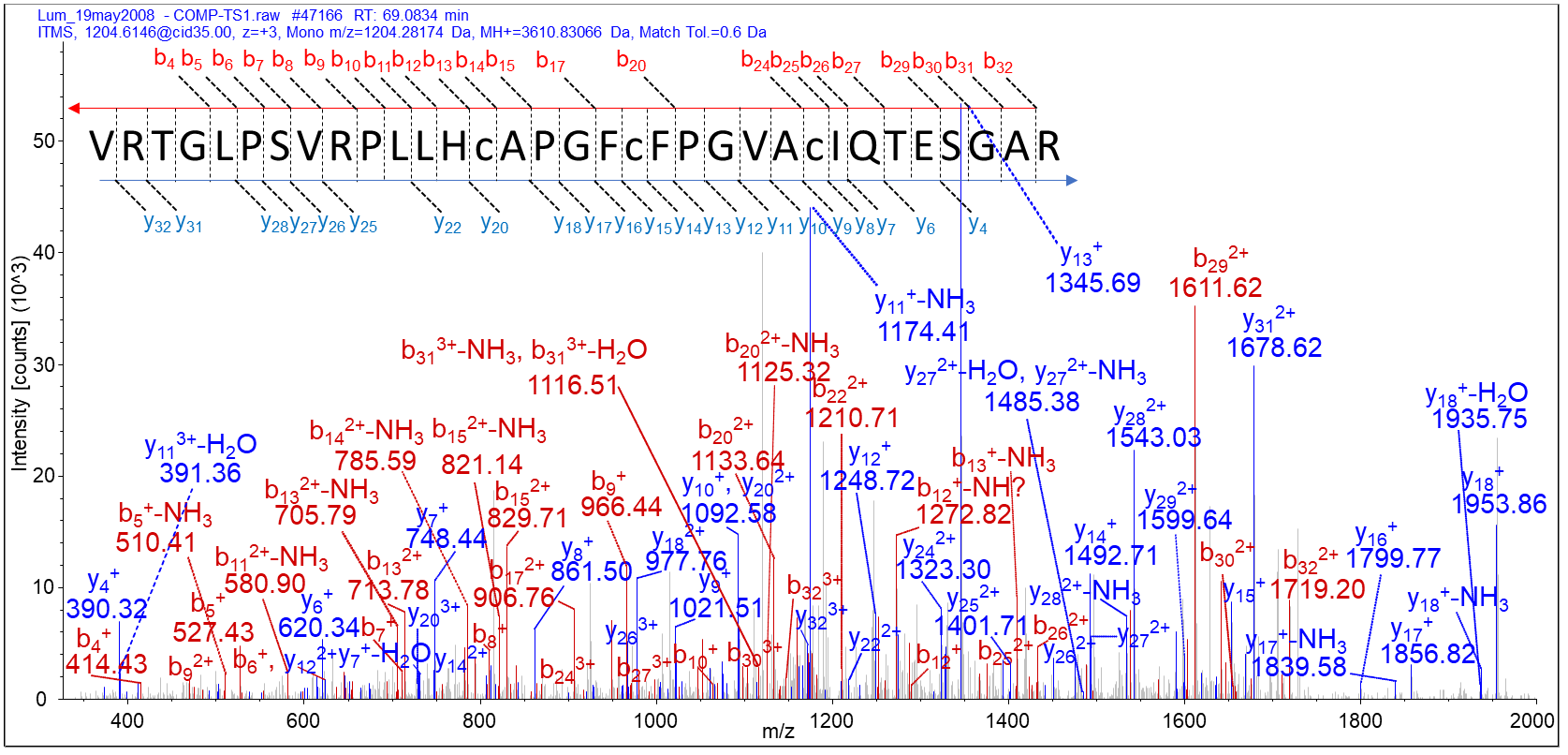
**

**
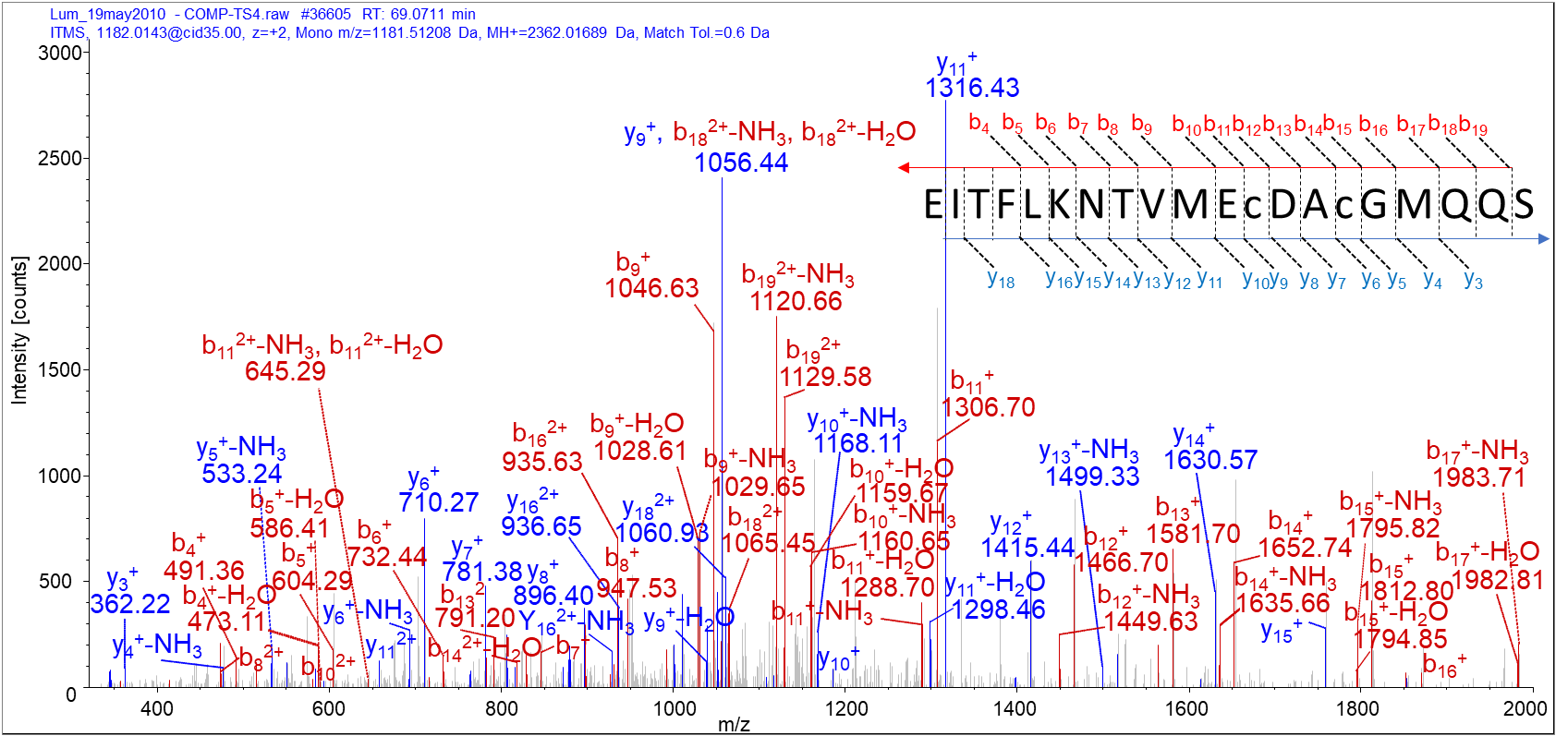
**

**
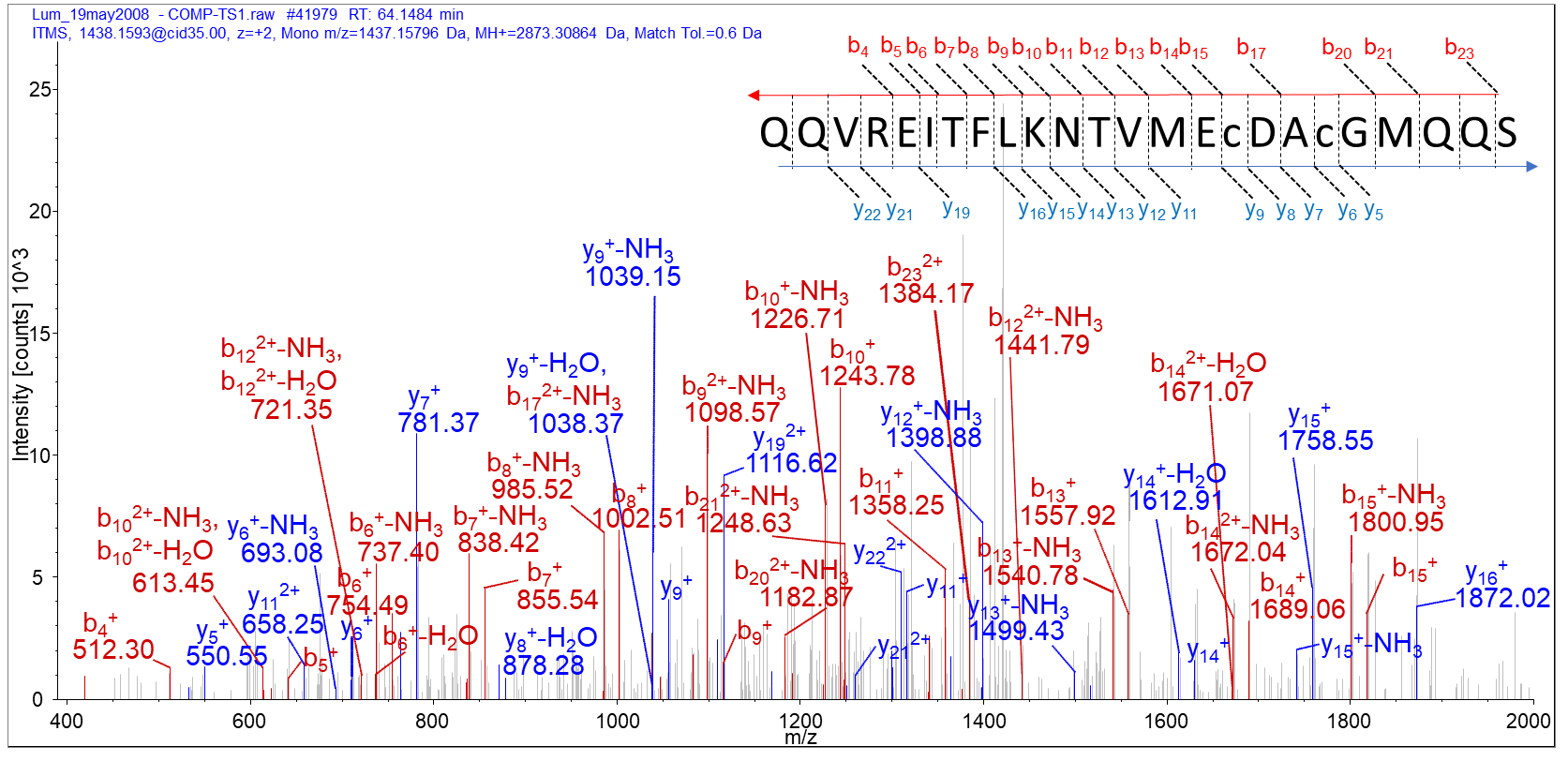
**

**Supplementary Figure 5. MMP1, 3, and 7 do not cleave COMP at the S^77^-V^78^ bond.** hCOMP (520 nM) was incubated with ADAMTS4 WT, MMP1, 3, 7 (each at 10 nM) or buffer alone (0) for 24 h at 37 ℃. Samples were then subjected to SDS-PAGE under non-reducing conditions and probed with a neoepitope anti-QQS^77^ polyclonal anti-COMP antibody. CF, cleavage fragment; CM, cleaved monomer, IB, immunoblot. Blot representative of two independent experiments.


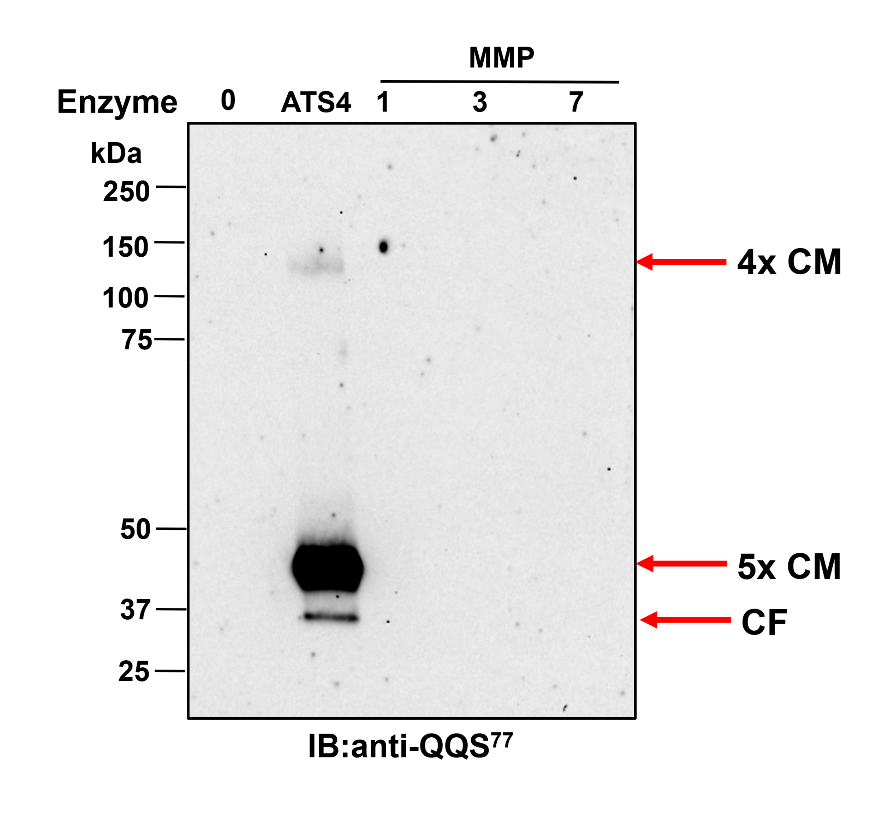


**Supplementary Figure 6.** Coomassie Brilliant Blue (CBB) staining (0.5 µg/lane) of purified

ADAMTS4 β3-β4; β9-β10 under reducing (5% β-mercaptoethanol) conditions. The red arrow indicates the mature, proteolytically active enzyme following prodomain removal.


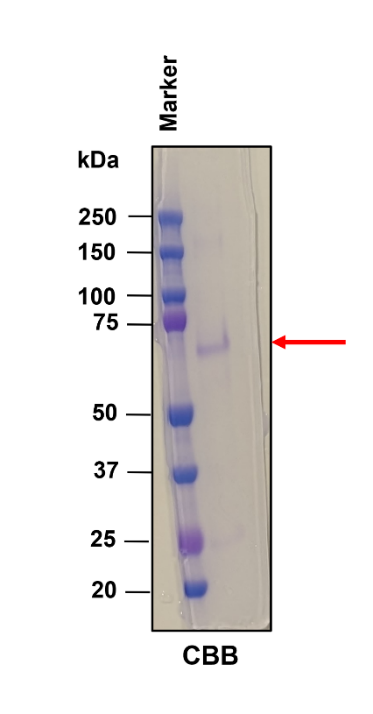


**Supplementary Figure 7.** Coomassie brilliant blue staining (1 µg/lane) of purified rec7E8.1E3 under non-reducing conditions. Red arrow indicates full-length IgG.

**
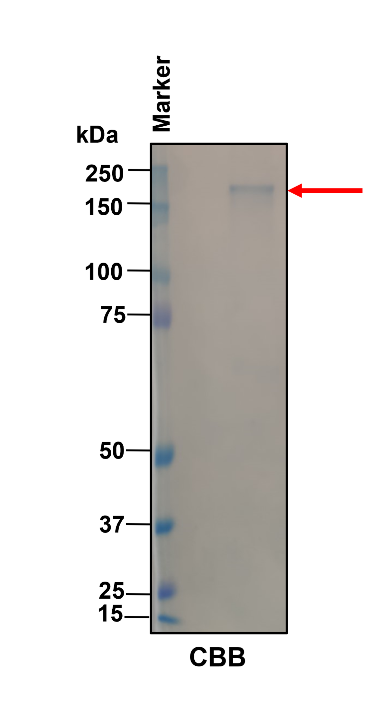
**

**Supplementary Figure 8. Selective inhibition of ADAMTS4 does not affect total COMP degradation in human OA cartilage explants.** The cartilage explants were dissected from the knee joints of OA patients (P3, P4) and rested in DMEM containing 10% FBS for 24 h. The medium was replaced, and the cartilage was rested for a further 24 h in DMEM at 37 °C before assays. The cartilage was further incubated with rec7E8.1E3 (αATS4, 10 and 100 nM) or isotype control (C, 100 nM). After 24 h incubation, the medium was collected, precipitated with trichloroacetic acid, dissolved in SDS sampling buffer and subjected to SDS-PAGE under non-reducing conditions followed by immunoblotting analysis with a polyclonal anti-COMP antibody. The black arrows indicate non-specific bands detected by the secondary antibody and matching the size of full-length IgGs (~150 kDa) and heavy chains (~50 kDa), respectively. D, dimer; I, immunoblot; M, monomer; P, pentamer; T, trimer.


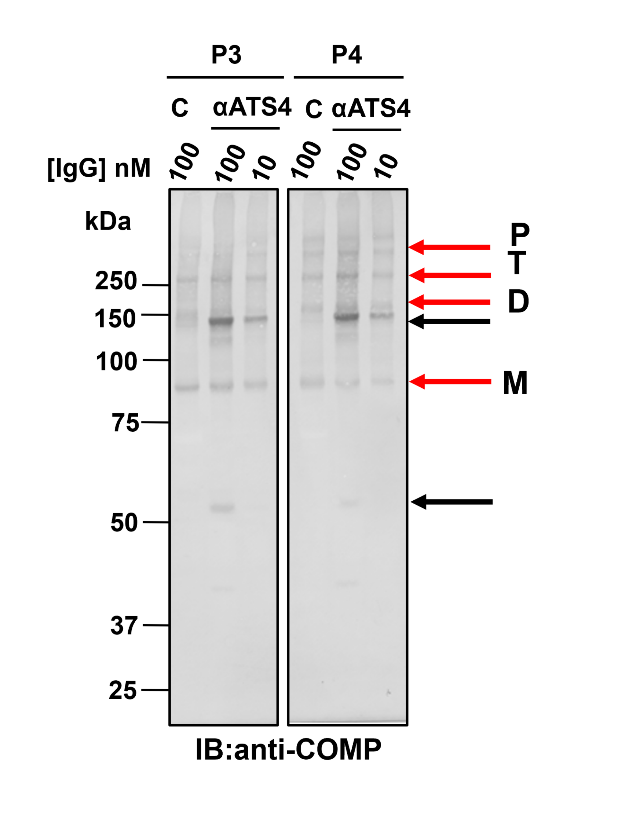


**Supplementary Figure 9. Cleavage site specificity of ADAMTS4.** IceLogo analysis of the cleavage site specificities of ADAMTS4 before (A) and after (B) inclusion of the newly identified COMP cleavage sites in the dataset. The height of each amino acid reflects its frequency in the specific positions between the various cleavage fragments as compared to the reference set whereas the colour represents its physicochemical properties. The cleaved scissile bond is represented by a dashed line and amino acid positions upstream and downstream of the cleavage site are numbered as unprimed and primed positions, respectively, according to the Schechter and Berger nomenclature.


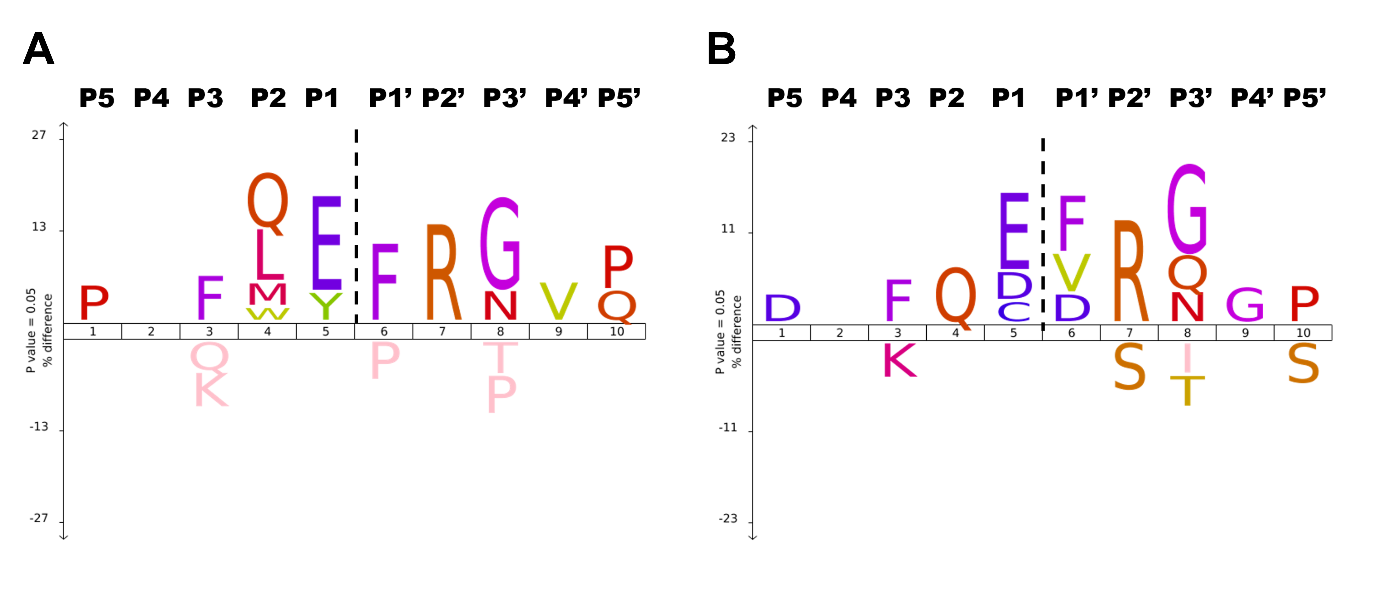


**Supplementary Figure 10:** Unprocessed gel for Figure 1B. Highlighted regions (in red box) corresponding to the data reported in the main manuscript figure.

**
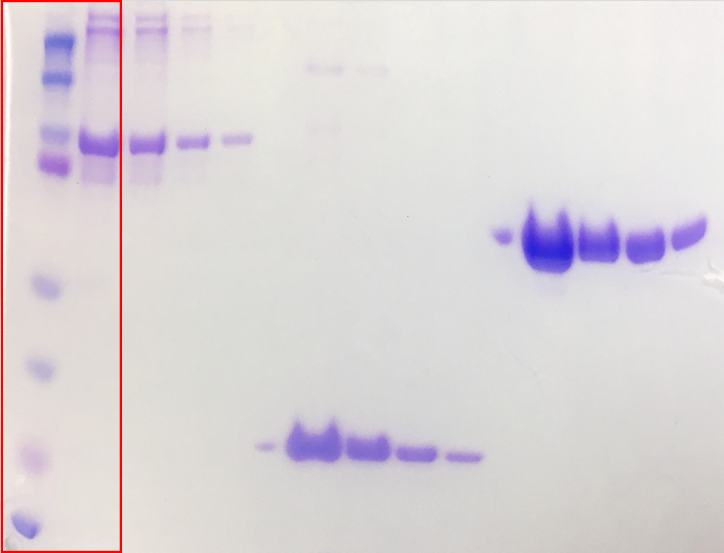
**

**Supplementary Figure 11:** Unprocessed gel for Figure 1C. Highlighted regions (in red box) corresponding to the data reported in the main manuscript figure.

**
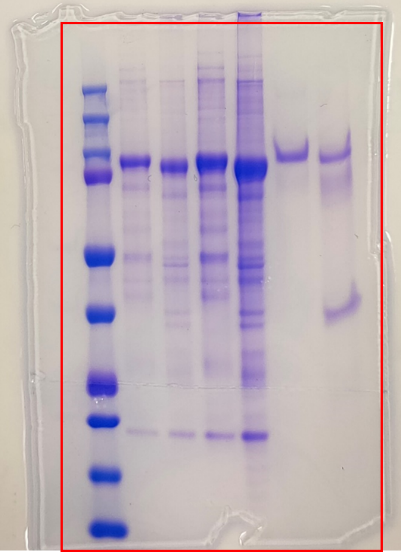
**

**Supplementary Figure 12:** Unprocessed gel for Figure 1D. Highlighted regions (in red box) corresponding to the data reported in the main manuscript figure.

**
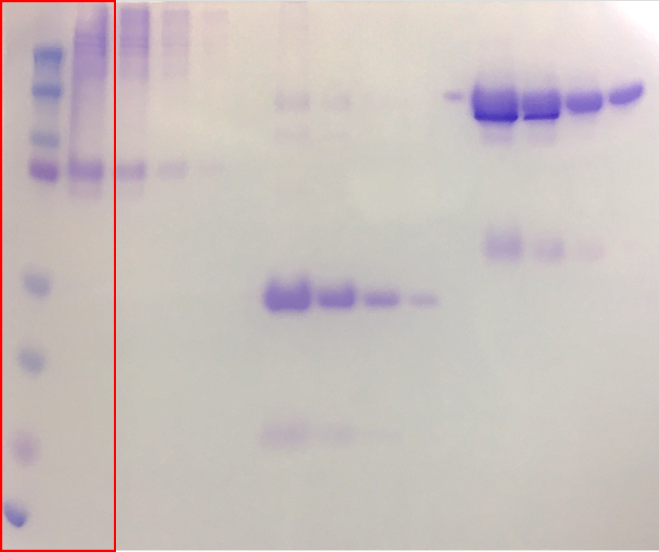
**

**Supplementary Figure 13:** Unprocessed gel for Figure 1E. Highlighted regions (in red box) corresponding to the data reported in the main manuscript figure.

**
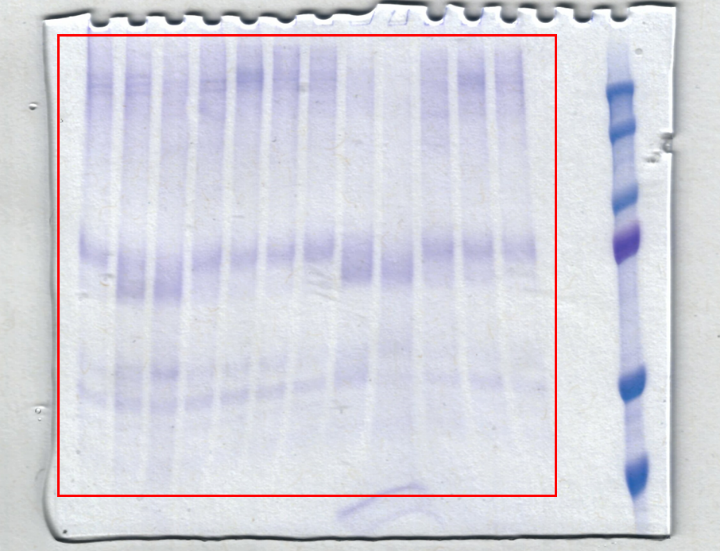
**

**Supplementary Figure 14:** Unprocessed gel for Figure 1F. Highlighted regions (in red box) corresponding to the data reported in the main manuscript figure.


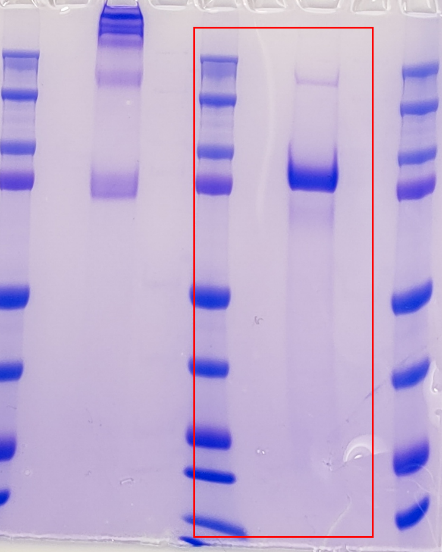


**Supplementary Figure 15:** Unprocessed gel for Figure 1G. Highlighted regions (in red box) corresponding to the data reported in the main manuscript figure.


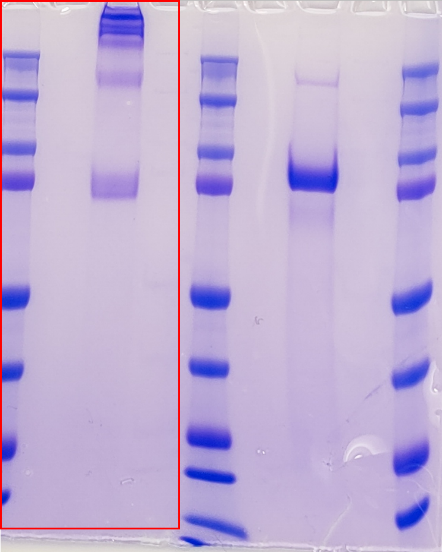


**Supplementary Figure 16:** Unprocessed immunoblot for Figure 1H. Highlighted regions (in red box) corresponding to the data reported in the main manuscript figure.


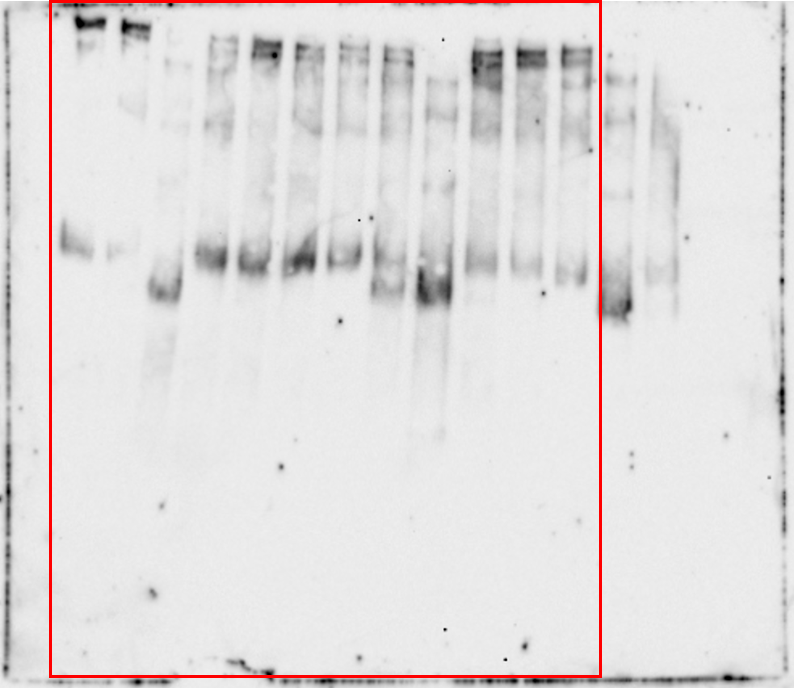


**Supplementary Figure 17:** Unprocessed immunoblot for Figure 1I. Highlighted regions (in red box) corresponding to the data reported in the main manuscript figure.


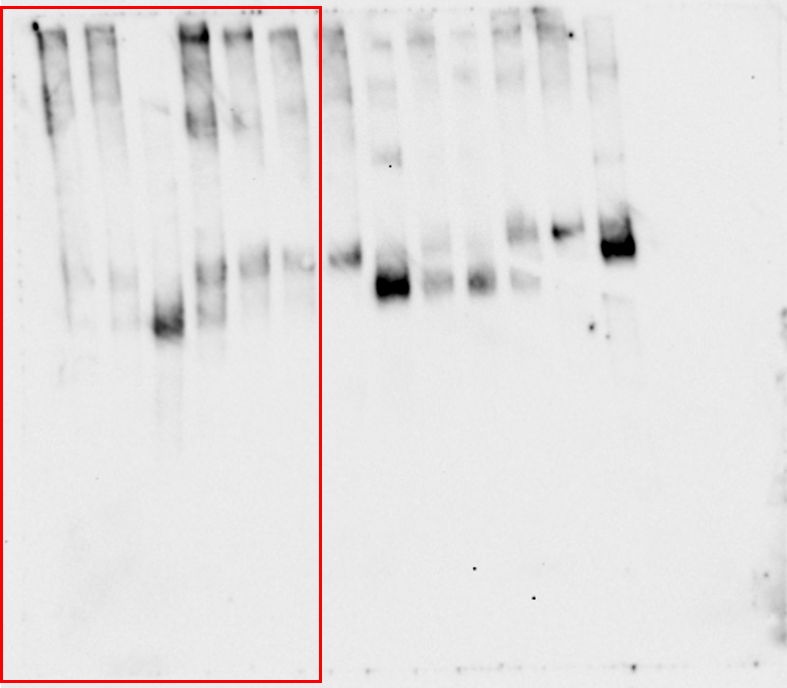


**Supplementary Figure 18:** Unprocessed immunoblot for Figure 1J. Highlighted regions (in red box) corresponding to the data reported in the main manuscript figure.

**
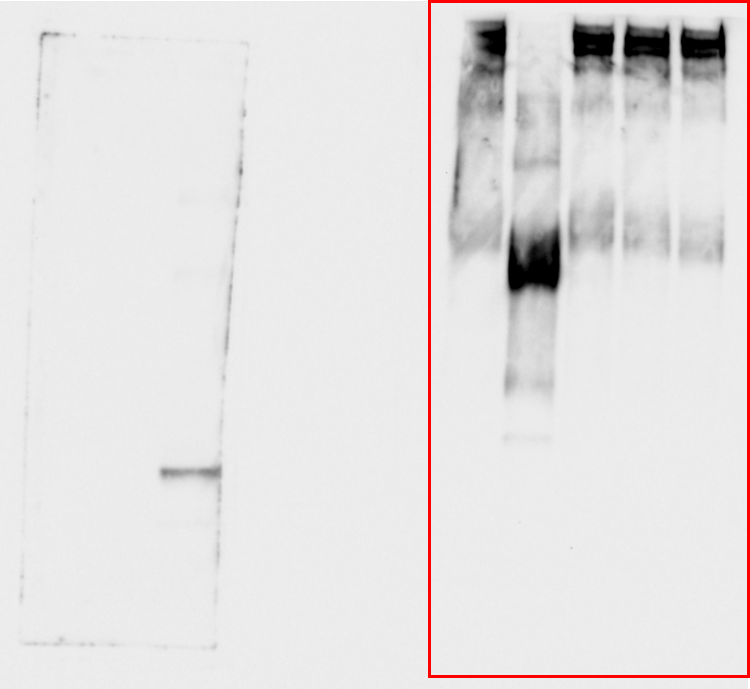
**

**Supplementary Figure 19:** Unprocessed immunoblot for Figure 2F. Highlighted regions (in red box) corresponding to the data reported in the main manuscript figure.

**
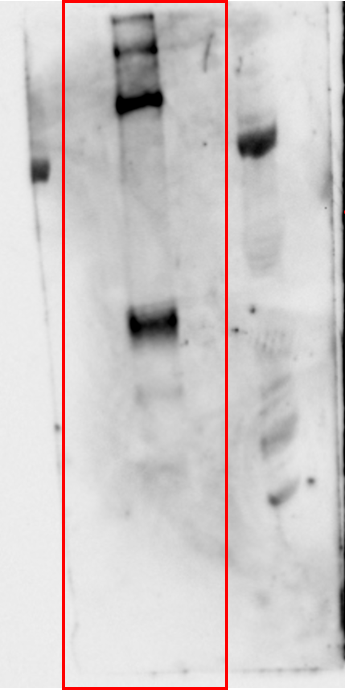
**

**Supplementary Figure 20:** Unprocessed immunoblot for Figure 2G. Highlighted regions (in red box) corresponding to the data reported in the main manuscript figure.

**
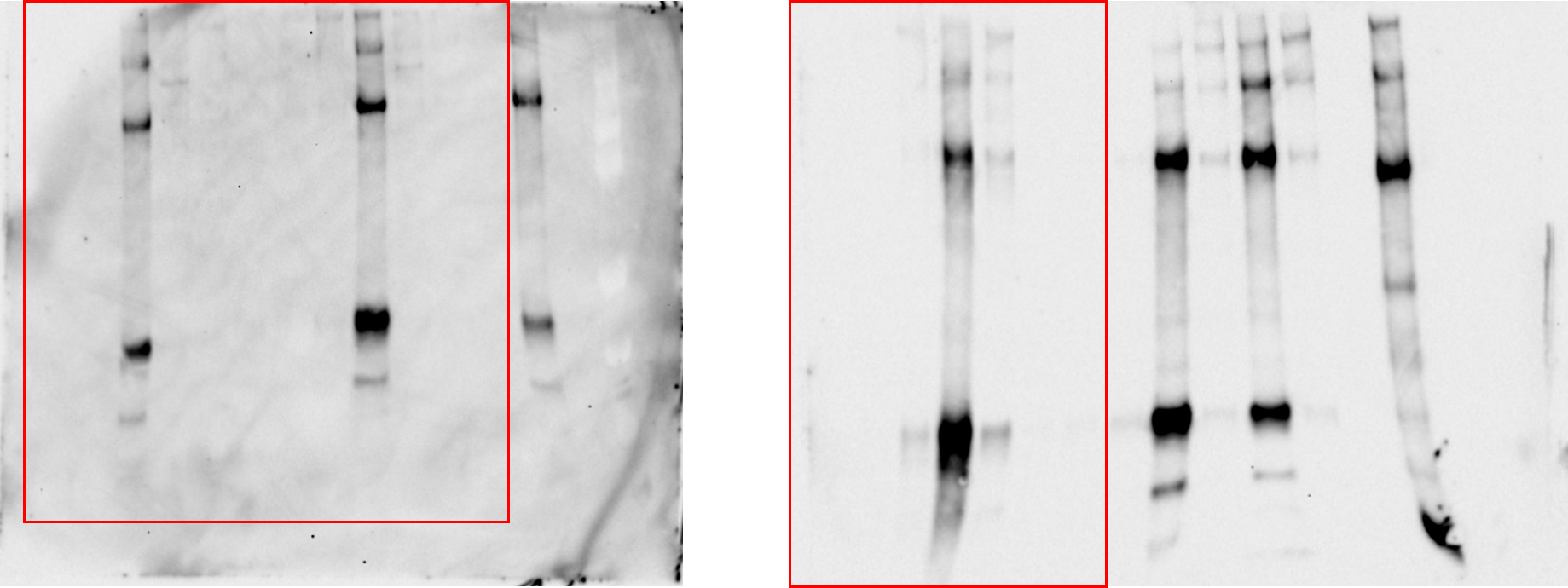
**

**Supplementary Figure 21:** Unprocessed immunoblot for Figure 3B. Highlighted regions (in red box) corresponding to the data reported in the main manuscript figure.

**
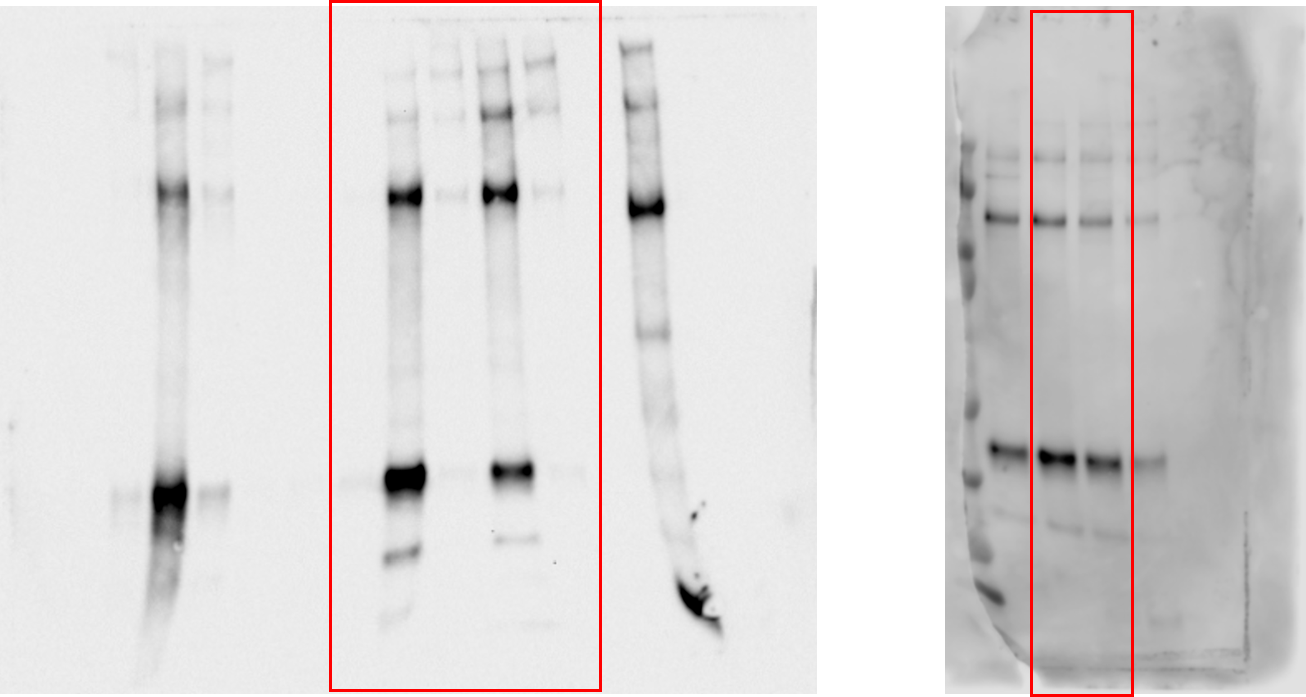
**

**Supplementary Figure 22:** Unprocessed immunoblot for Figure 5D. Highlighted regions (in red box) corresponding to the data reported in the main manuscript figure.


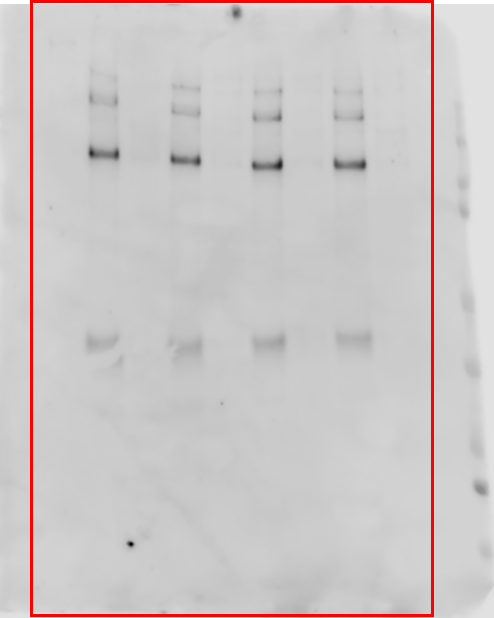


**Supplementary Figure 23:** Unprocessed immunoblot for Figure 5E. Highlighted regions (in red box) corresponding to the data reported in the main manuscript figure.

**
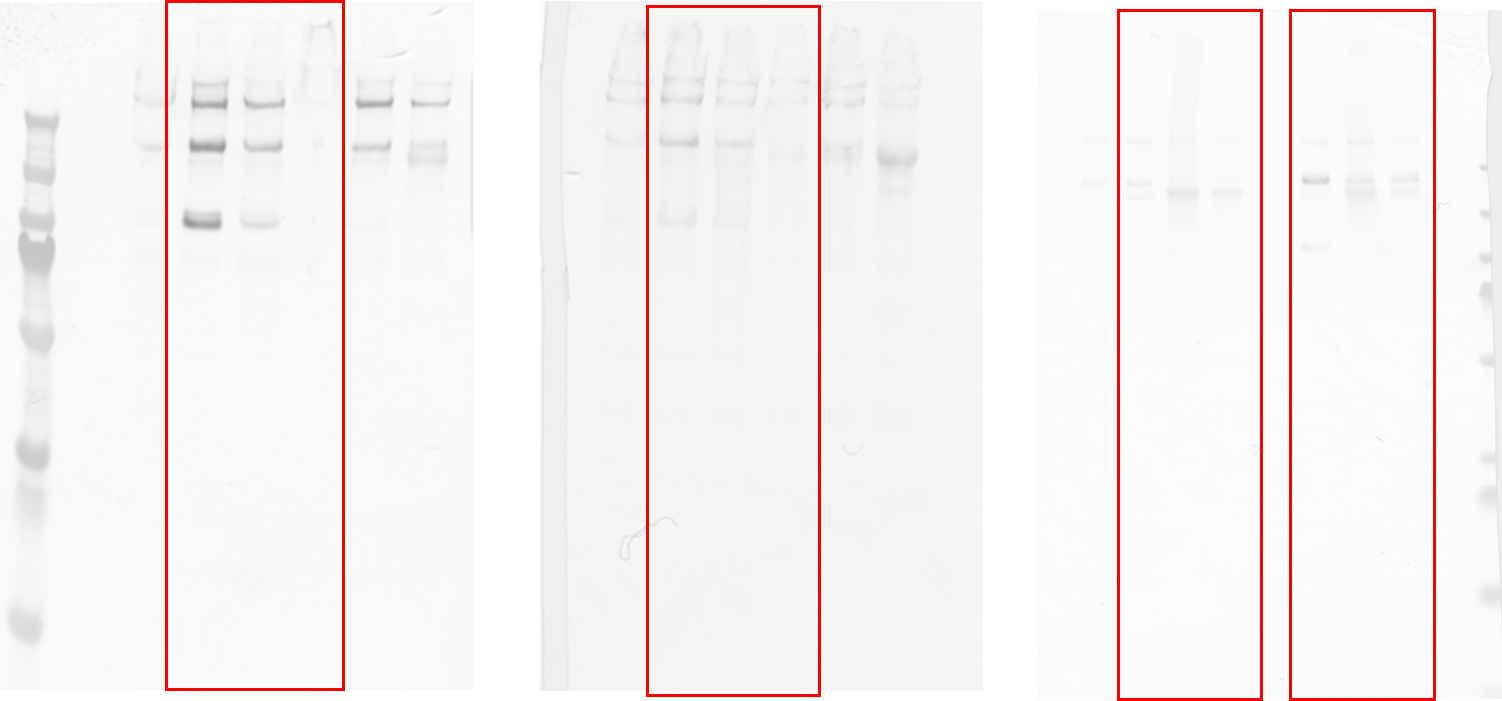
**

**Supplementary Figure 24. LDDT and PAE scores of the AlphaFold predicted complex between ADAMTS4 and pentameric COMP NTD.** (A) Local Distance Difference Test (LDDT) for the top 5 predicted models. B) Predicted Aligned Error (PAE) for the model with the highest score. C) Atomic coordinates of the model coloured according to LDDT (very high, LDDT>90, blue; confident, 90>LDDT>70, cyan; low, 70>LDDT>50, yellow; very low LDDT<50, orange).


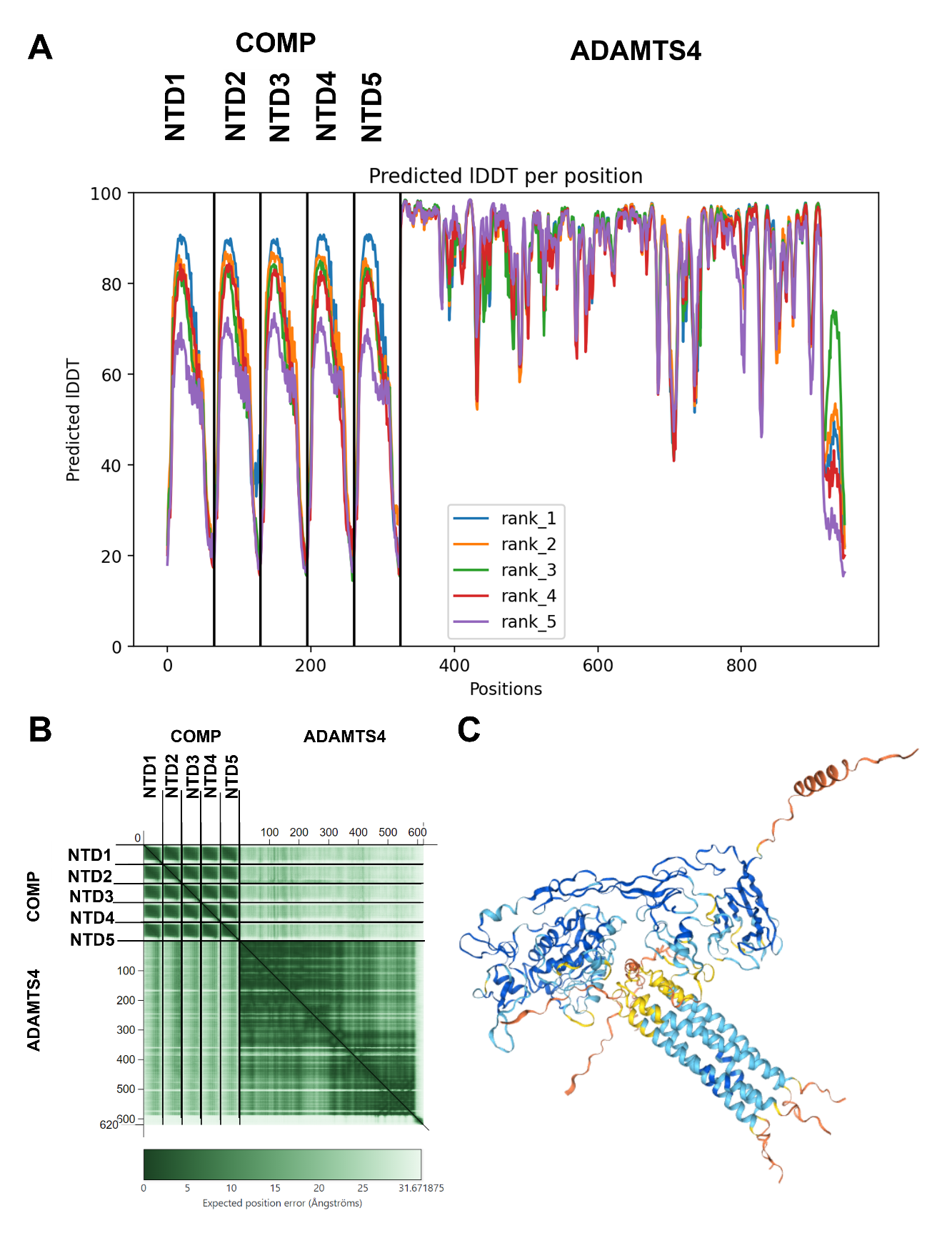

Supplement: De Groot 2025 Supplement [file NIHMS2099262-supplement-De_Groot_2025_Supplement.zip › 1-s2.0-S0945053X24001471-mmc2.docx]
